# Epitope-focused discovery of SARS-CoV-2 antibodies that potently neutralize Omicron variants

**DOI:** 10.1038/s41564-026-02282-x

**Published:** 2026-03-12

**Authors:** Seth J. Zost, Naveenchandra Suryadevara, Lauren E. Williamson, Suzanne M. Scheaffer, Elad Binshtein, Cameron D. Buchman, Nicole V. Johnson, Nicholas J. Catanzaro, Silvia Ravera, Nathaniel S. Chapman, Luke Myers, Ajit R. Ramamohan, Laura S. Handal, Doan C. Nguyen, Andrew Trivette, James R. Martinez, Eduardo Villalobos, Stacey A. Rutherford, F. Eun-Hyung Lee, Alexandra Schäfer, Ralph S. Baric, Jason S. McLellan, Michael S. Diamond, Robert H. Carnahan, James E. Crowe

**Affiliations:** 1 The Vanderbilt Center for Antibody Therapeutics, Vanderbilt University Medical Center, Nashville, TN USA; 2 Department of Pediatrics, Vanderbilt University Medical Center, Nashville, TN USA; 3 Department of Medicine, Washington University School of Medicine, StLouis, MO USA; 4 Department of Molecular Biosciences, The University of Texas at Austin, Austin, TX USA; 5 Department of Epidemiology, University of North Carolina at Chapel Hill, Chapel Hill, NC USA; 6 Department of Pathology, Microbiology, and Immunology, Vanderbilt University Medical Center, Nashville, TN USA; 7 Department of Medicine, Emory University, Atlanta, GA USA; 8 Department of Molecular Microbiology, Washington University School of Medicine, St. Louis, MO USA; 9 Department of Pathology and Immunology, Washington University School of Medicine, St Louis, MO USA; 10 The Andrew M. Jane M. Bursky Center for Human Immunology and Immunotherapy Programs, Washington University School of Medicine, St Louis, MO USA; 11 Center for Vaccines and Immunity to Microbial Pathogens, Washington University School of Medicine, St Louis, MO USA

**Keywords:** Antibodies, Cryoelectron microscopy

## Abstract

The emergence of SARS-CoV-2 Omicron variants has led to viral escape from many clinically approved monoclonal antibodies (mAbs) due to rapid evolution of the receptor-binding domain (RBD). Co-circulation of SARS-CoV-2 variants with unique sets of antigenic substitutions has further complicated therapeutic mAb discovery. New approaches are needed to rapidly discover and characterize mAbs with preferred specificity and functional characteristics. Here we describe and perform epitope-focused mAb discovery using glycan-masked antigens. We isolated and expressed a panel of 303 mAbs, some of which potently neutralize divergent Omicron subvariants by targeting the class 3 antigenic site on SARS-CoV-2 RBD. Epitope mapping of these antibodies revealed a spectrum of cross-reactivity and differential recognition of the class 3 site, validating the utility of this enrichment approach for targeted mAb discovery. Together, this work rationally designs glycan-masked engineered RBDs and uses them to isolate mAbs that potently neutralize antigenically divergent SARS-CoV-2 variants.

## Abstract (web summary)

The rapid evolution of SARS-CoV-2 challenges antibody therapeutics, but glycan-masked antigens enable isolation of class 3 monoclonal antibodies that potently neutralize diverse Omicron variants and reveal cross-reactive epitopes.

## Main

The COVID-19 pandemic spurred intense interest in human antibody responses to SARS-CoV-2, particularly neutralizing antibodies targeting the viral spike (S) glycoprotein. The trimeric S protein mediates viral entry and is the primary target of protective antibodies. Within S, the receptor-binding domain (RBD) is the dominant target of most potent neutralizing monoclonal antibodies (mAbs) identified so far. Many of these mAbs neutralize the virus by blocking the interaction between the RBD and the human receptor, angiotensin-converting enzyme 2 (hACE2).

**Fig. 1:**
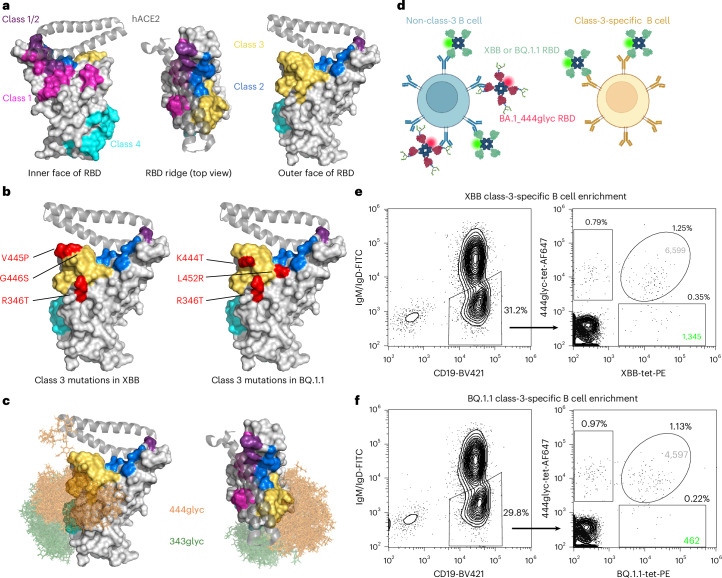
Epitope-focused approach for the enrichment of B cells specific for the SARS-CoV-2 RBD class 3 site. **a**, Views of the SARS-CoV-2 RBD and the locations of classes 1–4 are shown for the inner (cryptic) face (left), the hACE2 binding ridge from above (middle) and the outer face (right) of the RBD. The class 1 site is shown in magenta, class 2 site in blue, class 3 site in gold and class 4 site in cyan. Residues that overlap between classes 1 and 2 sites are shown in purple. Regions of hACE2 that interact with the RBD are represented in cartoon form (light grey). **b**, The outer face of the RBD with colouring as described in **a**. Class 3 site substitutions relative to BA.2 are indicated in red for XBB (left) or BQ.1.1 (right). **c**, Models of the RBD glycan conformations at positions 343 and 444. Thirty conformations are shown for each glycan (343 glycan in green, 444 glycan in orange). RBD structures with modelled glycans are coloured as described in **a** and shown from the outer face (left) and top orientation (right). **d**, Schematic representation of the sorting strategy to enrich for class-switched (IgM^−^/IgD^−^) B cells targeting the class 3 RBD site using BA.1-444 glycan-masked (444glyc) and XBB or BQ.1.1 RBD tetramers as antigen probes. **e**, Flow-cytometric enrichment strategy for the isolation of XBB-reactive B cells that failed to bind to the 444glyc RBD. **f**, Flow-cytometric enrichment strategy for isolation of BQ.1.1-reactive B cells that failed to bind to the 444glyc RBD. Numbers within gates indicate the total number of sorted events, and the percentage of events relative to the previous gate is also indicated. For all structures, a co-crystal structure of the RBD in complex with hACE2 (PDB ID: 6M0J) was used for visualization. Panel **d** created with BioRender.com.

Several classification schemes have been proposed to categorize RBD antibodies on the basis of their binding footprints and functional properties^1–4^. A widely used framework divides RBD-specific mAbs into four classes (classes 1–4) based on contact residues and modes of engagement^1,2^ (Fig. 1a). Class 1 mAbs bind epitopes that directly overlap with the hACE2 binding site and require the RBD to rotate into the ‘up’ conformation to bind the S trimer. Class 2 mAbs bind an opposing face of the RBD and can engage both open and closed conformations. Class 3 mAbs also recognize exterior RBD epitopes and bind both conformations, although only a subset directly block hACE2 binding^5^. Class 4 mAbs target cryptic epitopes at the base of the RBD and often require multiple RBDs to be in the open state. Potently neutralizing mAbs have been identified in classes 1–3, whereas class 4 mAbs generally exhibit lower neutralization potency. Several mAbs from classes 1–3 (refs. ^5–9^) were authorized for clinical use as COVID-19 prophylactics or therapeutics, including for prevention of symptomatic infection in immunocompromised individuals^10^.

Ongoing viral evolution has resulted in the emergence of SARS-CoV-2 variants with substantial antigenic changes. Omicron subvariants BA.1 and BA.2 contained 15 to 16 RBD substitutions relative to the ancestral Wuhan strain, many mapping to classes 1–4 antigenic sites, although the class 3 site was initially more conserved^3,11–15^. By late 2022, multiple variants with distinct combinations of class 3 mutations co-circulated. Highly immune-evasive variants, such as BQ.1.1 and XBB, each acquired three additional class 3 site substitutions relative to BA.2 (Fig. 1b), leading to loss of activity for many approved mAbs^16^. Despite antigenic drift, class 3 mAbs remain attractive therapeutic candidates. They often do not compete with class 1 mAbs for binding, reducing the likelihood that a single mutation would abrogate combination therapies^9,17^. In addition, synergistic neutralization has been observed for class 1/class 3 (refs. ^18,19^) and class 3/class 4 (ref. ^20^) mAb combinations, including improved activity of tixagevimab/cilgavimab against some Omicron variants^11,14^.

To identify new antibody candidates with functional efficacy against emerging variants, there remains a need for strategies to enrich for B cells encoding broadly protective antibodies targeting conserved antigenic sites, such as the class 3 site. Glycan masking, which introduces *N*-linked glycans to shield selected antigenic regions, has been used to generate epitope-focused immunogens, including of SARS-CoV-2 (refs. ^21–23^). Here we describe an antigenic-site-focused enrichment strategy using a glycan-masked RBD to isolate class-3-specific memory B cells cross-reactive with XBB or BQ.1.1 variants, followed by functional and structural characterization of the resulting mAbs.

## Results

### Epitope-focused mAb discovery approach to identify class-3-specific B cells

**Extended Data Fig. 1:**
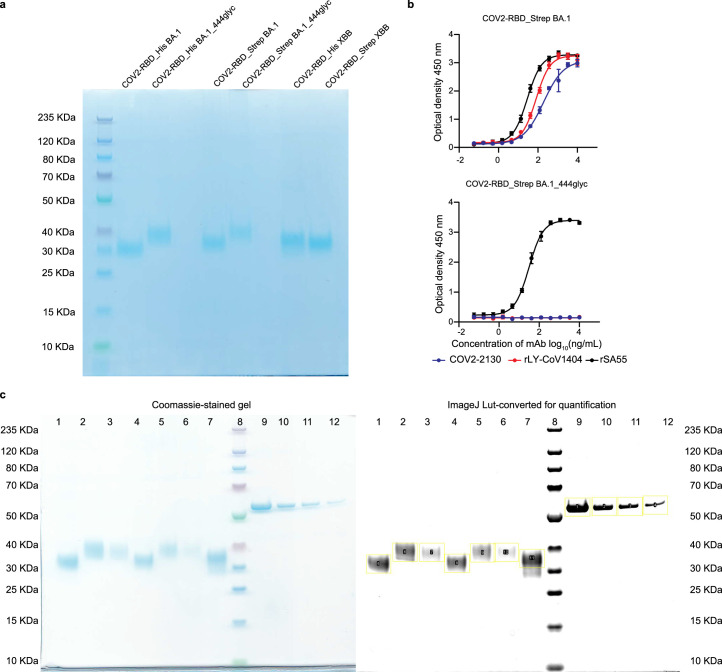
Glycan-masking validation of the BA.1 444-glyc RBD. **(a)** 4-12% SDS–PAGE of the COV2-RBD_His BA.1, COV2-RBD_His_BA.1_444glyc, COV2-RBD_Strep BA.1, COV2-RBD_Strep_BA.1_444glyc proteins. Shifts in molecular weight were observed for both antigen formats, indicating successful incorporation of the glycan. Gel run was performed twice (n = 2) **(b)** ELISA with COV2-RBD_Strep BA.1, COV2-RBD_Strep_BA.1_444glyc with class 3 mAbs COV2-2130 and rLY-CoV1404 and the positive control mAb rSA55. Data points shown are the mean ± SD of technical duplicates from a single representative experiment that was performed twice. **(c)** Coomassie stained 4-12% SDS–PAGE of BA.1 RBD proteins and standardized concentrations of BSA (left), ImageJ Lut-converted for quantification (right). Gel run was performed twice (n = 2). Band quantification values for respective proteins are available in Supplementary Table 1. Source data

We hypothesized that by glycan masking the class 3 site, we could use this antigen to enrich for B cells encoding mAbs reactive to this site through differential binding (Fig. 1c). We first generated a glycan-masked antigen by introducing a glycosylation sequon into the Omicron BA.1 RBD using K444N and S446T substitutions. In addition to being solvent accessible (a requirement for efficient glycosylation), K444 is an important contact residue for many previously described class 3 mAbs, including COV2-2130 (the parental mAb of cilgavimab) and LY-CoV1404 (the parental mAb of bebtelovimab)^7,9^. We confirmed that the BA.1_444glyc RBD (444glyc) shifted to a larger apparent molecular weight in SDS–PAGE than that of the native BA.1 RBD (Extended Data Fig. 1a,c,d), suggesting that this position within the class 3 site was glycosylated. COV2-2130 and a recombinant version of LY-CoV1404 (rLY-CoV1404) did not detectably bind this 444glyc antigen (Extended Data Fig. 1b). Next, we generated antigen probes for dual-colour antigen-specific B cell surface staining. To produce the probes, we biotinylated the 444glyc, XBB and BQ.1.1 RBDs and generated tetramers by coupling biotinylated antigens to fluorescently labelled streptavidin.

**Fig. 2:**
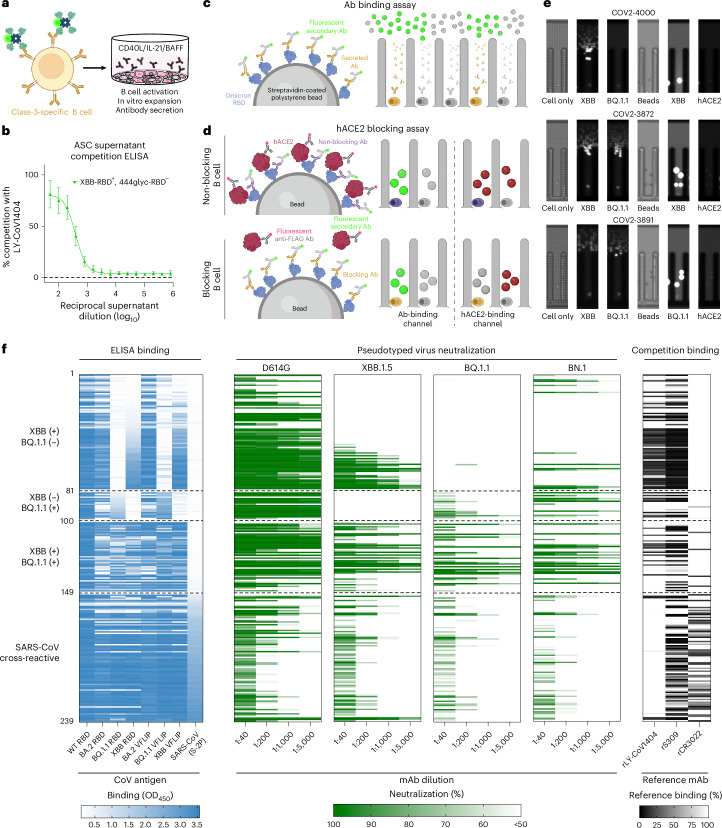
Isolation and initial characterization of RBD class-3-specific mAbs. **a**, Sorted memory B cells were stimulated in bulk in vitro on feeder layers expressing CD40L and secreting IL-21 and BAFF to generate ASCs. **b**, ASC culture supernatants collected from XBB-RBD^+^, 444glyc-RBD^−^ memory B cells were assessed for the ability to compete with the biotinylated class 3 site-specific reference mAb LY-CoV1404 for binding to BA.2 S_VFLIP antigen via a competition-binding ELISA. Competitive binding in the presence of supernatant from XBB-RBD^+^, 444glyc-RBD^−^ cells indicates successful enrichment for B cells targeting the class 3 site. Data points shown are the mean ± s.d. of ELISA signal from serial dilutions of ASC supernatants taken from wells with a single technical replicate per well (10 wells total). **c**, Schematic showing the Beacon instrument antibody-binding assay. Antibodies secreted by antigen-reactive B cells (yellow) bind to beads coated with an Omicron RBD, and secreted antibodies diffusing from within a pen are visualized as a plume of fluorescence signal (green). **d**, Schematic showing the Beacon hACE2 blocking assay. An antigen-reactive, non-blocking B cell (top, purple) shows signal in both the antibody-binding (green) and hACE2-binding channels (red), whereas an antigen-reactive, hACE2-blocking B cell (bottom, yellow) shows antibody-binding signal but little to no hACE2 signal. **e**, Beacon screening results for select B cells encoding 3 different mAbs. COV2-4000 (top) is XBB-reactive, BQ.1.1-non-reactive, and blocks binding of hACE2 to XBB. COV2-3872 (middle) is XBB- and BQ.1.1-reactive and blocks binding of hACE2 to XBB. COV2-3891 (bottom) is XBB- and BQ.1.1-reactive and blocks binding of hACE2 to BQ.1.1. **f**, A panel of ~300 mAbs was expressed recombinantly using a microscale expression platform, and the mAbs were tested for functionality. Left: the panel was tested for binding to RBDs from Wuhan-Hu-1 (WT), BA.2, XBB or BQ.1.1 variants and recombinant trimeric S proteins from BA.2, XBB, BQ.1.1 or SARS-CoV. A heat map displaying the optical density value at 450 nm (OD_450_, 0.05–3.5) for each antigen is indicated. White indicates a lack of detectable binding, whereas increasing depth of blue indicates higher OD values. The 239 mAbs shown have an OD of at least 0.5 against the WT RBD. Middle: neutralization potency of the panel was assessed against WA1/2020 D614G, XBB.1.5, BQ.1.1 or BN.1 pseudotyped viruses at 4 different dilutions of each mAb. A heat map of neutralization activity at each dilution is shown, where green indicates 100% neutralization and white indicates <50% neutralization. Right: epitope mapping of mAb panel by competition-binding assay with reference mAbs for class 3 (rLY-CoV1404, rS309) or class 4 (rCR3022). Binding of each reference mAb to trimeric BA.2_VFLIP antigen was measured in the presence of saturating concentrations of mAb in a competition-binding ELISA. Reference mAb binding was normalized to binding in the absence of competing antibody. The corresponding heat map indicates the extent of competition, with black representing 100% competition with the reference mAb and white representing 0% competition with the reference mAb. Binding, neutralization and competition-binding values are each derived from a single experiment. Panels **a**, **c** and **d** create with BioRender.com. Source data

**Extended Data Fig. 2:**
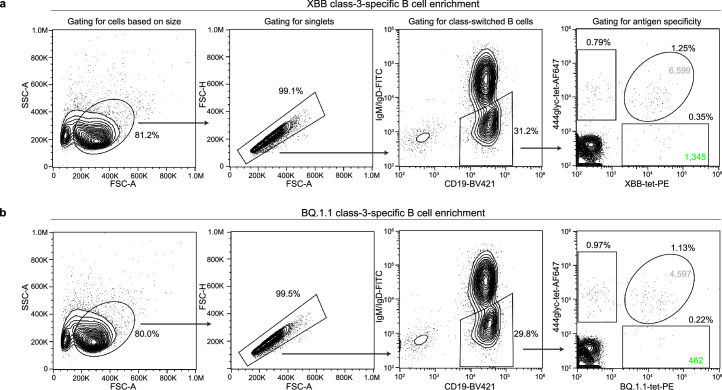
Representative gating strategies for flow-cytometric enrichment of class 3 site-specific B cells. Full flow-cytometric gating strategies for the antigen-specific sorting as shown in Fig. 1e, Fig. 1f and Fig. 6. Phenotypic markers are shown on each axis, and arrows indicate a cell population derived from a particular gate. Numbers indicate the percentage of each population relative to the total number of events within a given gate. **(a)** Gating strategy for enrichment of class 3 site-specific B cells that cross-react with the XBB RBD. **(b)** Gating strategy for enrichment of class 3 site-specific B cells that cross-react with the BQ.1.1 RBD. Numbers within gates indicate the total number of sorted events; the percentage of events relative to the previous gate is also indicated.

With these tools, we identified B cells from a participant (D2102) reactive to the class 3 RBD site through antigen-specific flow cytometry. We first enriched for B cells from peripheral blood mononuclear cells (PBMCs) using negative selection and then stained the B cells with antibodies specific for CD19, IgD and IgM. In addition to these B-cell phenotyping antibodies, we dual stained cells with the 444glyc-tet-AF647 and XBB- or BQ.1.1-tet-PE RBD tetramers (Fig. 1d). We defined the CD19^+^IgD^−^IgM^−^ population as class-switched memory B cells and identified antigen-reactive B cells from this population using RBD-tetramer staining (Fig. 1e,f and Extended Data Fig. 2). While XBB-RBD^+^, 444glyc-RBD^+^ cells were ~1.25% of total class-switched memory B cells, XBB-RBD^+^, 444glyc-RBD^−^ cells were present at an even lower frequency of ~0.35% (Fig. 1e). Of the class-switched memory B cells, ~0.79% were XBB-RBD^−^, 444glyc-RBD^+^, probably representing B cells that bound the BA.1 RBD containing the 444 glycan but did not cross-react with XBB. In comparison, BQ.1.1-RBD^+^, 444glyc-RBD^−^ cells were present at a frequency of ~0.22% (Fig. 1f). We defined the XBB or BQ.1.1-RBD^+^, 444glyc-RBD^−^ populations as class-3-specific B cells on the basis of the rationale that cells with this binding pattern would be shielded by the glycan (Fig. 1b,c). Using this enrichment strategy, we sorted ~1,300 XBB-RBD^+^ or ~460 BQ.1.1-RBD^+^, 444glyc-RBD^−^ B cells, and stimulated these cells in vitro to differentiate them into antibody-secreting cells (ASCs) (Fig. 2a), as previously described^24,25^. After 7 days in culture, we measured the ability of secreted antibodies in the ASC supernatants to compete for binding with the RBD class 3 mAb rLY-CoV1404. ASC supernatants from XBB-reactive class-3-specific B cells strongly (~80%) inhibited rLY-CoV1404 binding (Fig. 2b). The presence of rLY-CoV1404 blocking activity within the ASC supernatants indicated successful enrichment of class-3-specific B cells.

### Generation and initial characterization of class-3-specific RBD-targeting mAbs

After 9 days of expansion in culture, we separated ASCs from feeder layers for use in in two parallel workflows. We sequenced approximately half of the cells (~25,000) using the 10x Genomics Chromium single-cell sequencing platform, as previously described^25^. We loaded the remaining cells onto a Beacon optofluidic instrument to assess binding and functional activity of antibodies secreted from single B cells in individual pens. Employing this system, we measured antigen reactivity of secreted antibodies from thousands of individual B cells using an in-channel binding assay (Fig. 2c). We screened all B cells for reactivity to XBB or BQ.1.1 using consecutive in-channel assays. To identify individual B cells that might secrete neutralizing mAbs, we further assayed B cells for their ability to compete for binding with soluble hACE2 receptor to XBB or BQ.1.1 RBDs. To do this, we employed a previously developed in-pen blocking assay^25^ (Fig. 2d). In this assay, antibody binding to antigen-coated beads is measured simultaneously with hACE2 binding. B cells secreting a mAb that does not block hACE2 binding are identified by beads that are bright in both the antibody and hACE2 fluorescence channels, whereas B cells secreting a mAb that blocks hACE2 binding have beads that are bright in the antibody-binding fluorescence channel but dim in the hACE2 channel (Fig. 2d). From these antigen-binding and receptor-blocking assays, we identified B cells secreting mAbs that strongly inhibited the RBD–hACE2 interaction (Fig. 2e). After completing binding and blocking assays, we exported and sequenced the antibody variable region genes in XBB- and/or BQ.1.1-RBD-reactive B cells, prioritizing those that showed hACE2 blocking activity (Fig. 2e).

After recovery of paired heavy- and light-chain variable gene sequences from both workflows, we used a previously described high-throughput approach^25,26^ to express, purify and characterize a panel of 303 mAbs (Fig. 2f and Supplementary Table 2). We first tested these mAbs for binding in enzyme-linked immunosorbent assay (ELISA) to a panel of recombinant monomeric RBDs (Wuhan-1, BA.2, XBB and BQ.1.1), and trimeric S proteins of SARS-CoV-2 (BA.2, XBB and BQ.1.1) and SARS-CoV. Of these 303 mAbs, 239 reacted to SARS-CoV-2 Wuhan-1 (WT) RBD. Additional ELISA binding data revealed that the antibodies could be grouped on the basis of binding pattern reactivity to XBB, BQ.1.1 and SARS-CoV (Fig. 2f). In addition to binding assays, we also performed neutralization assays using a panel of 4 different lentiviral pseudotyped viruses (displaying S proteins of WA/1/2020 D614G, XBB.1.5, BQ.1.1 and BN.1 viruses) to further group and rank mAbs on the basis of neutralization potency and cross-reactivity. These data revealed that many of these mAbs potently neutralized either XBB.1.5 or BQ.1.1 at all tested dilutions, including a subset of mAbs that potently inhibited pseudotyped virus infection by both variants (Fig. 2f). We included the BN.1 variant because it contains an additional set of antigenic substitutions at R346T, K356T (which introduces an *N*-linked glycan at N354) and F490S, which are all in or near the class 3 antigenic site^27^. Some mAbs that potently neutralized XBB.1.5 or BQ.1.1 did not detectably neutralize BN.1, suggesting that differences in contact residues result in differential sensitivity to mutations. To map the relative binding sites of the mAb panel, we performed competition-binding studies with recombinant versions of reference antibodies known to recognize the class 3 site (rLY-CoV1404 (ref. ^7^) and rS309 (ref. ^5^)) or class 4 site (rCR3022 (refs. ^28,29^)) on the RBD (Fig. 2f). Of the mAb panel, 74 mAbs showed at least 33% competition with rLY-CoV1404 and 145 mAbs showed at least 33% competition with rLY-CoV1404 and rS309 for binding to BA.2 trimeric S protein, further supporting the successful enrichment for mAbs with class 3 site specificity using this approach. Many of the mAbs with the most potent neutralization activity against XBB.1.5 or BQ.1.1 competed with rLY-CoV1404 for binding. Antibody competition with either rS309 or rCR3022 was enriched among SARS-CoV cross-reactive mAbs, consistent with these mAbs recognizing epitopes conserved between SARS-CoV-2 and SARS-CoV.

### Comprehensive characterization of the binding and neutralization profiles of downselected class-3-specific mAbs

**Fig. 3:**
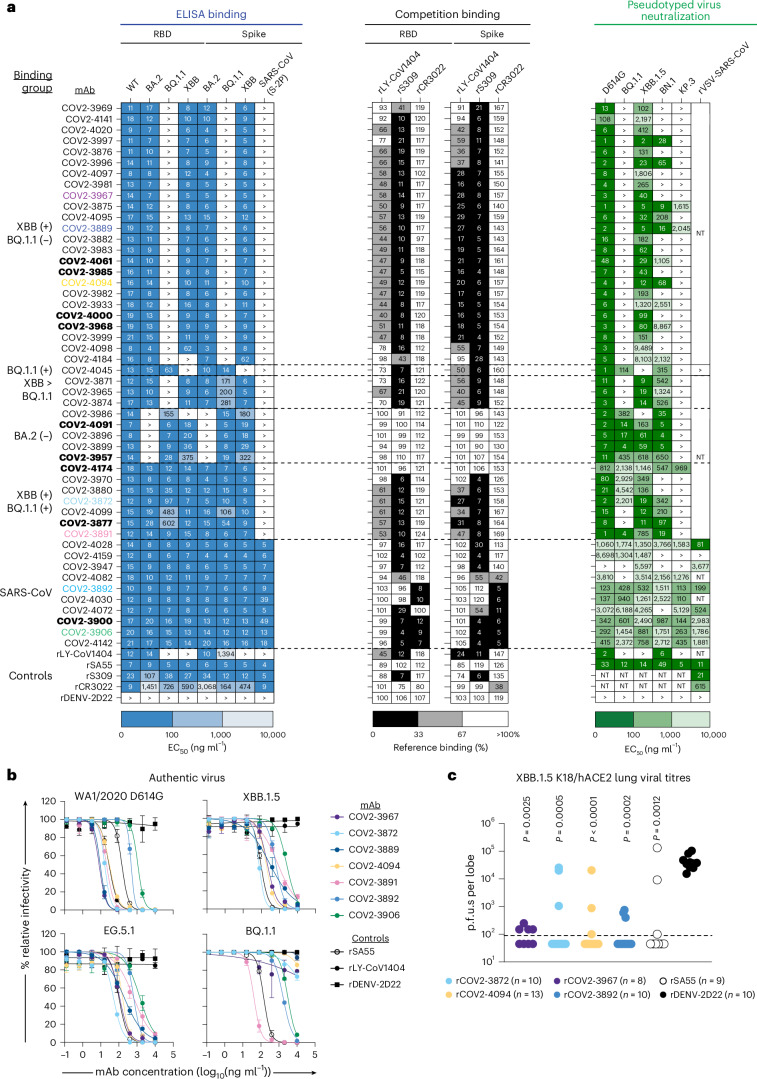
Downselection of class-3-site-enriched mAbs, confirmation of authentic virus neutralization and protection from infection. **a**, Left: representative EC_50_ values for ELISA binding reactivity to Wuhan-Hu-1 (WT), BA.2, BQ.1.1 or XBB recombinant monomeric RBD (left) and BA.2, XBB or BQ.1.1 VFLIP, or SARS-CoV (S2P) recombinant trimeric S proteins (right). The heat map corresponds to EC_50_ values < 100 (dark blue), >100 to 1,000 (blue) or >1,000 to 10,000 (light blue) ng ml^−1^ as indicated. The > symbol indicates EC_50_ values greater than 10,000 ng ml^−1^. On the basis of ELISA binding reactivity, mAbs were classified into different binding groups, XBB (+) BQ.1.1 (−), BQ.1.1 (+), XBB > BQ.1.1, BA.2 (−), XBB (+) BQ.1.1 (+) and SARS-CoV cross-reactive. Positive (rLY-CoV1404, rSA55, rS309, rCR3022) and negative (rDENV-2D22) control mAbs are indicated accordingly. Data are representative of 2 independent experiments performed in technical triplicates. Middle: representative percent binding of mAbs in the presence of reference class 3 (rLY-CoV1404, rS309) or class 4 (rCR3022) mAbs to recombinant BA.2 monomeric RBD (left) or trimeric S protein (VFLIP; right) via competition-binding ELISA. Relative maximal signal for binding was normalized to binding in the presence of the isotype-matched negative control mAb rDENV-2D22. Per cent binding was then calculated, and the resulting values are shown. The heat map highlights mAbs in which full (black; <33%), intermediate (grey; 33–67%) or no (white; >67%) competition was observed in the presence of the corresponding reference mAb. Data are representative of percent binding values from 2 independent experiments performed in single technical replicates. Right: representative IC_50_ values for neutralization activity against pseudotyped viruses WA1/2020 D614G, BQ.1.1, XBB.1.5, BN.1, KP.3 or rVSV-SARS-CoV. The heat map corresponds to IC_50_ values < 100 (dark green), >100 to 1,000 (green) or >1,000 to 10,000 (light green) ng ml^−1^ as indicated. The > symbol indicates IC_50_ values greater than 10,000 ng ml^−1^; NT, not tested. Data are representative of 2 independent experiments performed in technical duplicate. MAbs further selected for authentic SARS-CoV-2 neutralization assays or negative-stain electron microscopy studies (Extended Data Fig. 5) are bolded or coloured, respectively, for reference. **b**, Representative neutralization curves for selected mAbs against authentic SARS-CoV-2 virus strains (WA1/2020 D614G (top left), XBB.1.5 (top right), EG.5.1 (bottom left) and BQ.1.1 (bottom right)). Positive (rSA55, open circles) and negative (rLY-CoV1404, closed circles; rDENV-2D22, closed squares) control mAbs also were included. Each curve displays mAb concentration (log_10_(ng ml^−1^)) on the *x* axis and percent relative viral infectivity on the *y* axis. Data shown are mean ± s.d. of technical duplicates and are representative of 2 independent experiments. Representative neutralization curves for the full downselected panel of 16 mAbs are shown in Extended Data Fig. 3. **c**, Passive transfer of SARS-CoV-2 mAbs prevents viral replication in vivo. Lung viral titres in p.f.u. per lobe from K18-hACE2 transgenic mice that received mAb 1 day before challenge with an authentic XBB.1.5 viral isolate. Data points represent lung titres from individual mice; the dashed line indicates the lower limit of quantification. Group sizes are indicated in the figure legend. *P* values are derived from the comparison of each group to the rDENV-2D22 isotype-matched mAb control group using a non-parametric Kruskal–Wallis test with Dunn’s post hoc correction for multiple comparisons. *P* = 0.0025 (COV2-3967), *P* = 0.0005 (COV2-3872), *P* < 0.0001 (COV2-4094), *P* = 0.0002 (COV2-3892) and *P* = 0.0012 (rSA55). Data shown are from 2 independent experiments. Source data

From the panel of 239 WT RBD-reactive mAbs, we downselected to a smaller subset of 50 mAbs for further characterization. These mAbs were chosen on the basis of a combination of cross-reactivity, competition binding, neutralization potency and gene usage. We completed quantitative dose–response binding assays to determine binding strength or half-maximal effective concentration (EC_50_) values relative to the previously described panel of antigens (Fig. 3a, left). On the basis of binding reactivity, we stratified mAbs into different groups. These groups were further assessed to map relative binding sites, via competition-binding studies with the reference antibodies for class 3 (rLY-CoV1404, rS309) or class 4 (rCR3022) (Fig. 3a, middle), to recombinant BA.2 monomeric RBD and trimeric S protein.

Most mAbs (*n* = 24) reacted with XBB but not with BQ.1.1 monomeric RBD or trimeric S protein at the concentrations tested (XBB (+) BQ.1.1(−)). Several mAbs (*n* = 3) exhibited stronger binding reactivity to XBB than BQ.1.1 and preferentially bound trimeric S protein (XBB > BQ.1.1). Of the downselected panel, only one mAb reacted to BQ.1.1 (BQ.1.1(+)) but did not react to XBB monomeric RBD or trimeric S protein. Based on competition-binding studies, these mAbs predominantly competed with rS309 on monomeric RBD, and rS309 and rLY-CoV1404 on the trimeric S protein.

The remaining mAbs (*n* = 7) cross-reacted with the SARS-CoV-2 antigens tested (Wuhan-1 [WT], BA.2, XBB, BQ.1.1; XBB (+) BQ.1.1 (+)) and could be further grouped on the basis of cross-reactivity to SARS-CoV (*n* = 10). Similar to the XBB- or BQ.1.1-reactive mAbs, the XBB and BQ.1.1 cross-reactive mAbs predominantly competed with rS309 and less so with rLY-CoV1404. Consistent with the binding profile of the SARS-CoV-reactive mAbs, most of these mAbs competed with the class 4 site reference mAb rCR3022 and the class 3 site reference mAb rS309.

We next performed quantitative dose–response neutralization assays using pseudotyped viruses displaying the S protein of WA1/2020 D614G, BQ.1.1, XBB.1.5, BN.1 and the more antigenically distinct variant KP.3 to determine half-maximal inhibitory concentration (IC_50_) values for neutralization (Fig. 3a, right). While the neutralization activity corresponded with the binding profiles to SARS-CoV-2 monomeric RBD antigens, there were differences in binding strength and neutralization potency. A loss of neutralization activity was observed for some mAbs to BN.1, which is consistent with viral escape from some class 3 mAbs due to BN.1 containing substitutions near the class 3 antigenic site. However, several mAbs maintained moderate neutralization activity against BN.1. For the SARS-CoV-reactive mAbs, we also assessed neutralization activity against a recombinant vesicular stomatitis virus (VSV) expressing the SARS-CoV S protein (rVSV-SARS-CoV). These mAbs exhibited neutralization activity that was moderate (<81 ng ml^−1^ IC_50_ value) to weak (<3,677 ng ml^−1^ IC_50_ value) against rVSV-SARS-CoV, consistent with their binding and neutralization activity against SARS-CoV-2 variants. From the set of mAbs that competed with rLY-CoV1404 and rS309, COV2-3875 and COV2-3889 weakly neutralized a KP.3 pseudotyped virus. Given that these mAbs are both encoded by *IGHV2-26* and *IGKV1-39* (Supplementary Table 2), it is likely that they recognize the class 3 antigenic site in a way that partially tolerates the extensive class 3 substitutions present in KP.3 (V445H, G446S, N450D) in addition to changes adjacent to the class 3 site (K356T, L452W).

A small subset of XBB- and BQ.1.1-reactive mAbs (*n* = 5, that is, COV2-3896, -3899, -3957, -3986 and -4091) did not react to BA.2 and did not compete with the reference antibodies tested (rLY-CoV1404, rS309 or rCR3022). However, consistent with the binding profile to monomeric RBD, these mAbs neutralized the pseudotyped viruses tested (WA1/2020 D614G, BQ.1.1, XBB.1.5 and BN.1). This finding suggests that these mAbs (10% of the total panel) may recognize a site distinct from class 3 or 4.

### Class-3-site-specific mAbs potently neutralize authentic SARS-CoV-2

**Extended Data Fig. 3:**
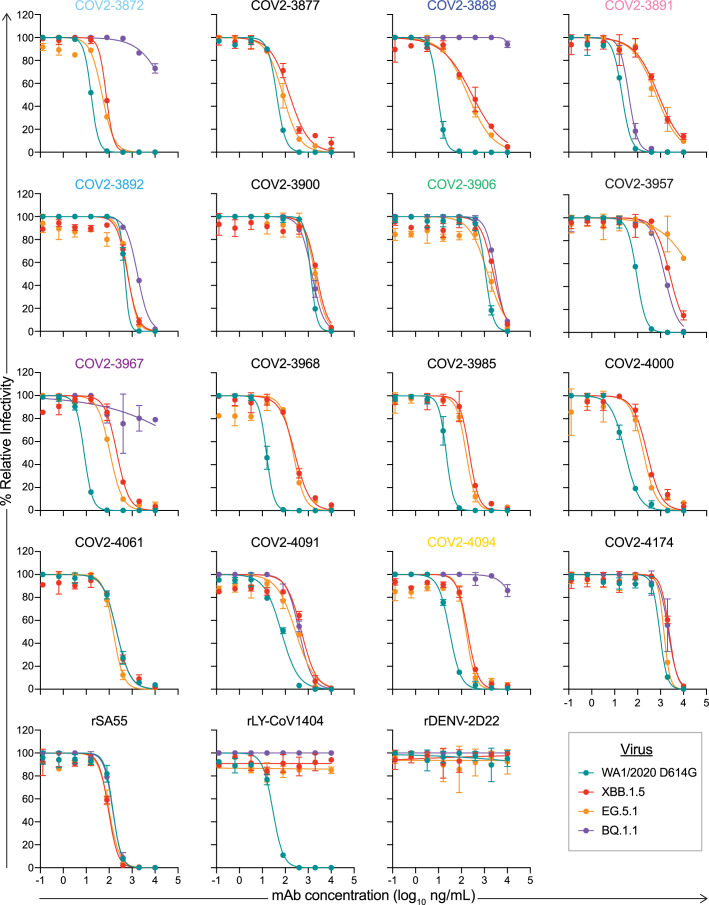
Neutralization curves for down-selected mAbs against authentic SARS-CoV-2 isolates. Representative neutralization curves for focus reduction neutralization tests (FRNT) assessing the ability of serial dilutions of mAbs to neutralize authentic WA1/2020 D614G (teal), XBB.1.5 (red), EG.5.1 (orange), or BQ.1.1 (purple) variants. Four-parameter sigmoidal dose-response curves were fit to the data using GraphPad Prism. MAbs that were selected for structural studies are shown in different colors. Data points shown are the mean ± SD of technical duplicates from a single representative experiment that was performed twice. The same neutralization data for some mAbs are also shown in Fig. 3b. Source data

On the basis of antibody binding and neutralization profiles, we further downselected 16 representative mAbs for assessment of neutralization activity against authentic SARS-CoV-2 viruses (D614G, XBB.1.5 and EG.5.1) (Fig. 3b, and Extended Data Fig. 3 and Table 1). A subset of mAbs selected on the basis of BQ.1.1 reactivity and structural characterization were also tested for neutralization activity against an authentic BQ.1.1 isolate. Consistent with the neutralization activity profiles against the pseudotyped viruses tested, these mAbs neutralized authentic SARS-CoV-2 in a similar manner, albeit with some differences in potency.

**Extended Data Fig. 4:**
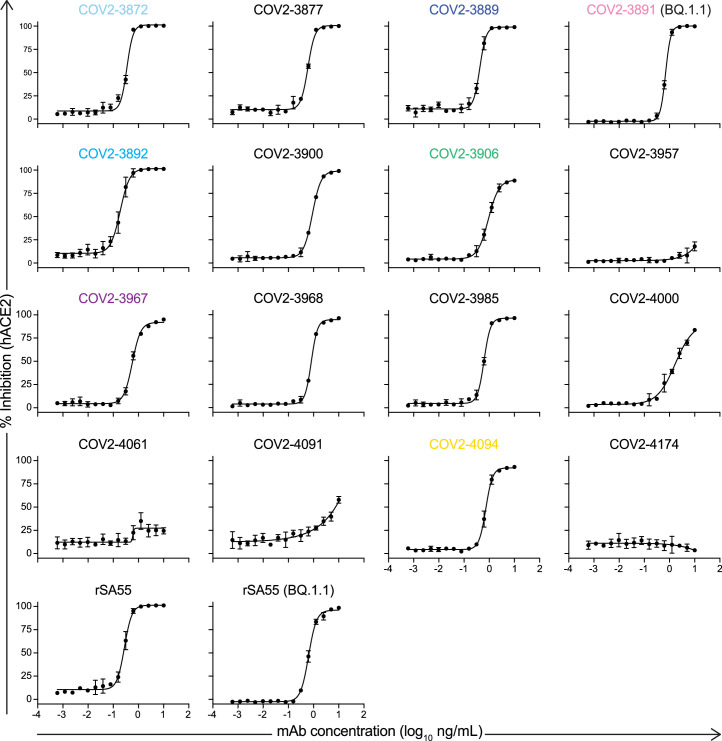
Receptor-blocking activity assessment of down-selected mAbs using a hACE2-blocking ELISA. Competition-binding ELISA measuring the ability of serial dilutions of mAbs to block binding of hACE2 to XBB S_VFLIP trimers (or BQ.1.1 as denoted in parentheses). Reductions in hACE2 binding were determined relative to the signal in wells that received no blocking mAb. Four-parameter sigmoidal dose-response curves were fit to the data using GraphPad Prism. MAbs that were selected for structural studies are shown in different colors. Data points shown are the mean ± SD of technical triplicates, except in the case of the positive control mAb rSA55, which was performed in technical duplicate. Data shown are from a single representative experiment that was performed twice. Source data

To determine the neutralization mechanism of action for these mAbs, we performed quantitative dose–response hACE2-blocking studies for binding to XBB or BQ.1.1 trimeric S proteins (Extended Data Fig. 4). Most of the mAbs tested completely blocked hACE2 binding. However, several mAbs partially (that is, COV2-3906, -4000 and -4091) or negligibly (that is, COV2-3957, -4061 and -4174) blocked hACE2 binding to the S protein at the concentrations tested.

### Class-3-site-specific mAbs reduce viral titre in mice

We next tested COV2-3872, -3892, -3967 and -4094 for in vivo protection in a SARS-CoV-2 XBB.1.5 mouse model using K18/hACE2 transgenic mice^30,31^. For the XBB (+) BQ.1.1 (−) mAbs, we selected both COV2-3967 and -4094 due to differences in BN.1 pseudotyped virus neutralization activity, while COV2-3872 or COV2-3892 were chosen as representative mAbs for XBB (+) BQ.1.1 (+) or SARS-CoV cross-reactive mAbs, respectively. To assess in vivo protective activity, 20–25-week-old K18-ACE2 mice were administered 200 µg of antibody via intraperitoneal injection 24 h before intranasal inoculation with 10^4^ plaque-forming units (p.f.u.s) of SARS-CoV-2 XBB.1.5. Three days after virus inoculation, lungs were collected and corresponding viral titres were determined via plaque assay (Fig. 3c). Based on these results, all mAbs (COV2-3872, -3892, -3967, -4094 and the positive control rSA55) significantly reduced lung viral titres compared to the negative control (rDENV-2D22).

### Structural studies confirm the enrichment of a diverse panel of class-3-site-specific mAbs

**Extended Data Fig. 5:**
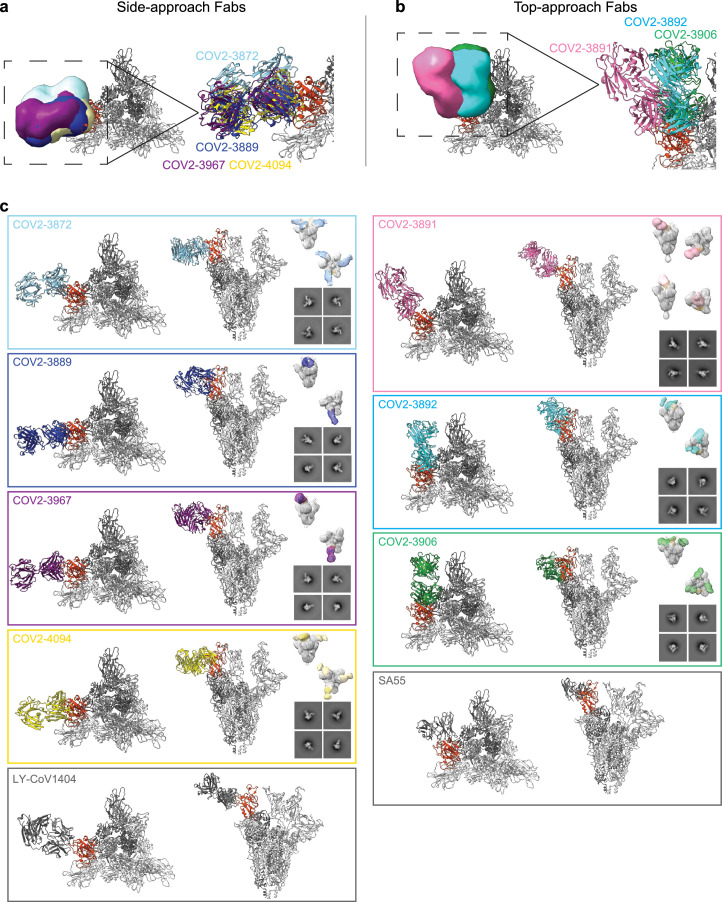
Negative-stain electron microscopy confirms the isolation of diverse class 3 site-specific mAbs. Negative-stain electron microscopy was performed to confirm that the enriched mAbs recognize the class 3 RBD site of XBB or BQ.1.1 (VFLIP) trimeric S protein. Two main groups were elucidated, in which Fab molecules bound the RBD from either the side (**a**) or top (**b**). **(a)** Fab molecules approaching from the side include COV2-3872 (light blue), −3889 (dark blue), −3967 (purple), and −4094 (yellow). An overlap representation of these Fab molecules for binding to the cartoon representation of the XBB trimeric S protein is shown (left). A zoomed-in view showing the binding of overlapping Fab molecules to the RBD site (right). **(b)** Fab molecules approaching from the top include COV2-3891 (pink), −3892 (cyan), and −3906 (green). An overlap representation of these Fab molecules for binding to the cartoon representation of the XBB or BQ.1.1 (COV2-3891) trimeric S protein is shown (left). A zoomed-in view showing the binding of overlapping Fab molecules to the RBD site (right). **(c)** Individual representations are indicated for each corresponding mAb separated out by the appropriately colored box. In each box, cartoon representations of Fab molecule binding to the RBD site of the trimeric S protein from the top- (left) or side-view (middle) perspective. Representative 3D reconstruction maps (top right) and 2D class average images (bottom right) are also shown. Fab molecules that approach from the side or top are on the left- or right-hand side, respectively. Reference class 3 site-specific mAb LY-CoV1404 and class 1 site-specific mAb SA55 are included to provide points of reference for viewing Fab molecule binding to the class 3 RBD site and relative angles of approach.

On the basis of binding and neutralizing activity profiles of representative group members and unique antibody variable gene usages, we selected 7 mAbs to confirm their relative binding site to SARS-CoV-2 XBB or BQ.1.1 trimeric S protein (VFLIP) by negative-stain electron microscopy (Extended Data Fig. 5 and Supplementary Table 3). Two main mAb groups were defined on the basis of the general binding angle of the Fab molecules relative to the antigen. Full occupancy was observed for COV2-3872, -3906 and -4094, in which a Fab molecule was bound to each of the three RBDs on the trimeric S protein. In contrast, partial occupancy was observed for COV2-3892 or COV2-3889, -3891 and -3967 with 2 or 1 Fab molecules, respectively, bound to the S trimer. The angle of binding of COV2-3892 and COV2-3906 is consistent with their competition for binding with the class 4 mAb rCR3022, suggesting that the epitope of these mAbs overlaps with the class 4 site.

**Fig. 4:**
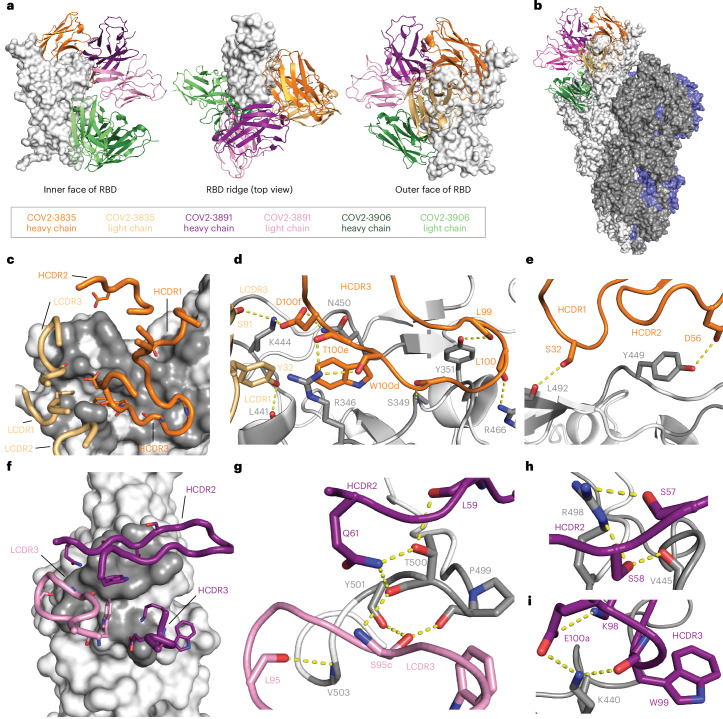
Cryo-EM reconstructions of COV2-3835 and COV2-3891 Fab molecules bound to the spike RBD. **a**, Visualization of COV2-3835, -3891 or -3906 Fab molecules with different orientations of the RBD (that is, inner face (left), hACE2 binding ridge (middle) and outer face (right)), shown by the white molecular surface representation. The determined structures of COV2-3835 and COV2-3891 were aligned to the XBB.1.5 RBD:COV2-3906 Fab complex (PDB ID: 9C6Y). The Fv regions of COV2-3835, -3891 and -3906 Fab molecules are shown as cartoon representations with the corresponding colour coding: COV2-3835 heavy chain in orange, COV2-3835 light chain in gold, COV2-3891 heavy chain in purple, COV2-3891 light chain in pink, COV2-3906 heavy chain in dark green, and COV2-3906 heavy chain in light green. COV2-3835 and COV2-3891 bind to the class 3 antigenic site, while COV2-3906 binds the class 4 antigenic site. **b**, The Fv regions of COV2-3835, -3891 and -3906 Fab molecules aligned to an RBD in the open conformation of the SARS-CoV-2 spike trimer (PDB ID: 6VSB). Protomers of the SARS-CoV-2 spike trimer are shown in white, grey and blue. **c**, The CDRs of COV2-3835 are overlaid on the COV2-3835 epitope, with RBD contact residues coloured in grey. **d**,**e**, A detailed view of COV2-3835 CDR interactions with the BA.1 spike protein. Putative hydrogen bonds are represented by yellow dashed lines. **f**, The CDRs of COV2-3891 are overlaid on the COV2-3891 epitope, with RBD contact residues in grey. **g**–**i**, Detailed views of COV2-3891 HCDR2 and LCDR3 interactions with T500, Y500, and V503 of the BQ.1.1 spike protein (**g**) of COV2-3891 HCDR2 interactions with V445 and R498 of the BQ.1.1 spike protein (**h**) and detailed view of COV2-3891 HCDR3 interactions with K440 of the BQ.1.1 spike protein (**i**).

**Fig. 5:**
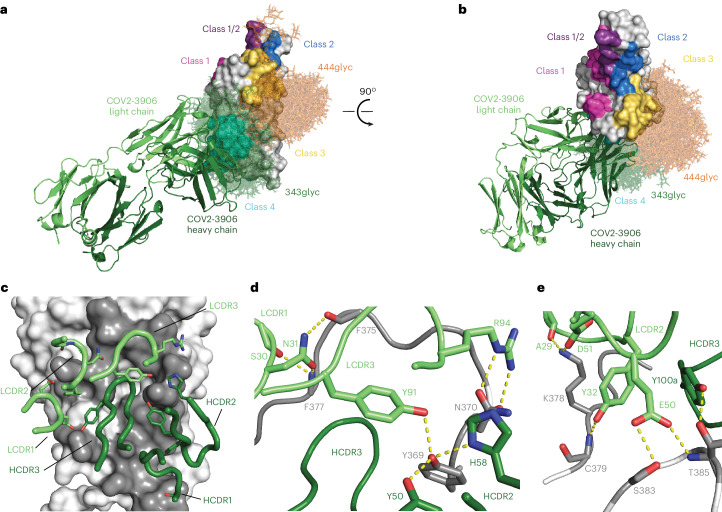
Structure of the XBB.1.5 RBD:COV2-3906 Fab complex as determined by X-ray crystallography. **a**,**b**, Visualization of the COV2-3906 epitope from the side (**a**) and top (**b**) view of the XBB.1.5 RBD. Antigenic sites are labelled and coloured as described in Fig. 1. COV2-3906 binds to the class 4 site (cyan). **c**, The CDRs of COV2-3906 are overlaid on the COV2-3906 epitope, with the RBD contact residues shown in grey. **d**, A detailed view of the interactions of COV2-3906 HCDR2 and LCDR3 interactions with the XBB.1.5 RBD. **e**, A detailed view of COV2-3906 HCDR3 and LCDR2 interactions with the XBB.1.5 RBD. Putative hydrogen bonds are represented by yellow dashed lines.

**Extended Data Fig. 6:**
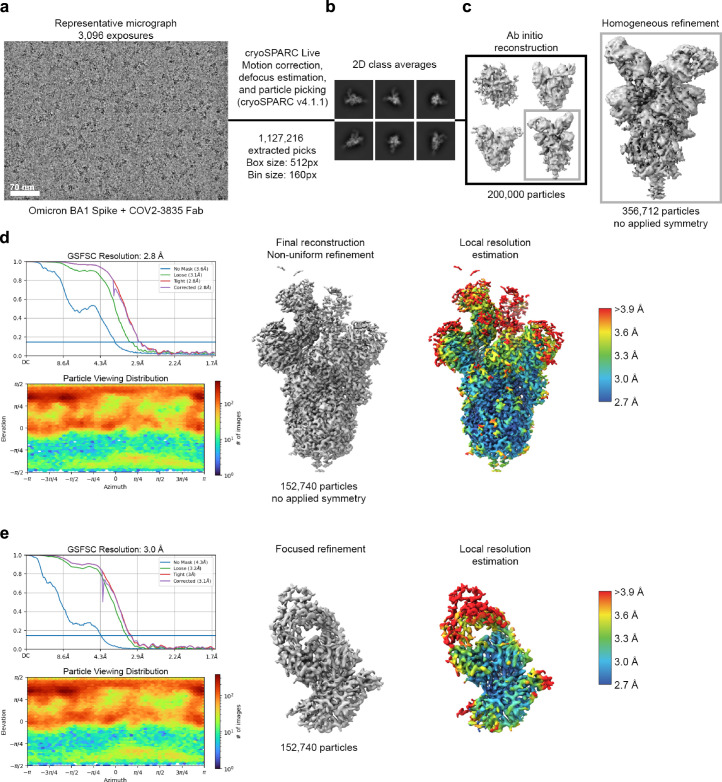
Cryo-EM data processing workflow of the BA.1 spike protein in complex with COV2-3835 Fab molecules. **(a)** Representative electron micrograph of the BA.1 spike protein bound by COV2-3835 Fab molecules embedded in vitreous ice. **(b)** 2D class averages of the BA.1 spike: COV2-3835 Fab molecules complex. **(c)** *Ab initio* reconstruction (left), and homogeneous refinement (right) of the global EM map. **(d)** Fourier shell correlation curves and particle orientation distribution (left), EM map (middle) and EM map colored by local resolution (right) for the full spike protein:Fab molecule reconstruction. **(e)** Fourier shell correlation curves and particle orientation distribution (left), EM map (middle) and EM map colored by local resolution (right) for focused refinement of the RBD:COV2-3835 Fab interaction. For Fourier shell correlations, the 0.143 cutoff is indicated by a blue horizontal line.

**Extended Data Fig. 7:**
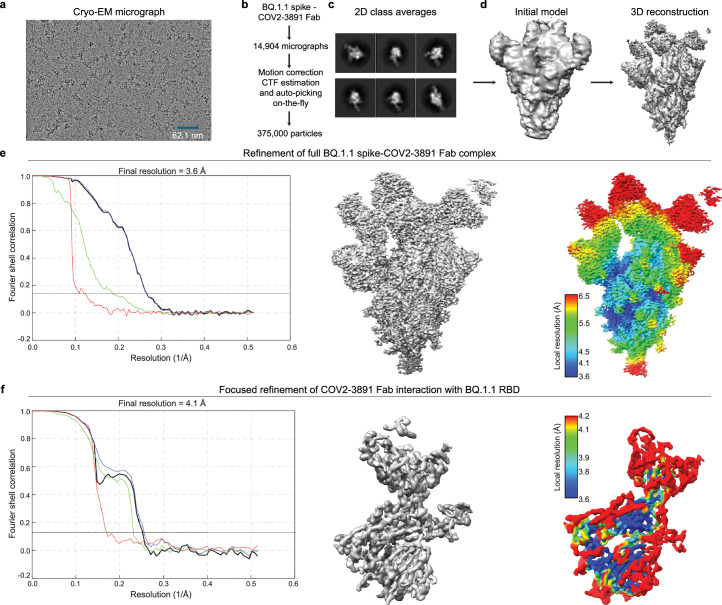
Cryo-EM data processing workflow of the BQ.1.1 spike protein in complex with COV2-3891 Fab molecules. **(a)** Representative electron micrograph of the BQ.1.1 spike protein bound by COV-3891 Fab molecules embedded in vitreous ice. **(b)** Sample pre-processing workflow. **(c)** 2D class averages of the BQ.1.1 spike:COV2-3891 Fab complex. The box sizes of the 2D class average images are ~388 Å. **(d)** Initial model (left), and 3D reconstruction (right) for the EM map. **(e)** Fourier shell correlation curves (left), EM map (middle) and EM map colored by local resolution (right) for the full spike protein:Fab molecule reconstruction. **(f)** Fourier shell correlation curves (left), EM map (middle) and EM map colored by local resolution (right) for focused refinement of the RBD:COV2-3891 Fab interaction. For Fourier shell correlations, the 0.143 cutoff is indicated by a horizontal dashed line. Red curves denote phase-randomized masked data, green curves denote raw data, blue curves denote masked data, and black curves denote corrected data. Local resolution maps were calculated with Relion. CTF: contrast transfer function.

We next sought to use higher-resolution approaches to better understand mAb binding and began by examining a 3.0 Å cryogenic electron microscopy (cryo-EM) reconstruction of a previously identified antibody, COV2-3835, in complex with the BA.1 spike (Fig. 4a–e, Extended Data Fig. 6 and Supplementary Table 3). COV2-3835 is a member of the same clonotype as COV2-3876, -3882, -3999 and -4000, all of which compete with both rLY-CoV1404 and rS309, neutralize XBB.1.5, but fail to neutralize BQ.1.1 or BN.1 (Fig. 3 and Supplementary Table 2). In addition, we determined a 4.1 Å cryo-EM reconstruction of COV2-3891 Fab molecules in complex with the BQ.1.1 S_VFLIP protein (Fig. 4a,b,f–i and Extended Data Fig. 7) and a 2.7 Å crystal structure of COV2-3906 Fab molecules in complex with the XBB.1.5 RBD (Figs. 4a,b and 5). These structures confirmed that COV2-3835 and COV2-3891 bind the class 3 site, whereas COV2-3906 binds the class 4 site (Fig. 4a,b).

Examination of the heavy- and light-chain complementarity-determining regions (CDRs) of COV2-3835 in contact with the BA.1 RBD revealed that COV2-3835 makes extensive contacts with residues within the class 3 antigenic site (Fig. 4d). Residues S91 and D100f (Kabat numbering) in the LCDR3 and HCDR3 of COV2-3835, respectively, form hydrogen bonds with K444. D100f also interacts with N450, while HCDR3 residues W100d and T100e form hydrogen bonds with R346. W100d may also form a cation–pi interaction between the aromatic ring of the tryptophan and the positive charge of the R346 side chain. Residues in the HCDR1 and HCDR2 of COV2-3835 also mediate additional contacts with L492 and Y449, respectively (Fig. 4e). BQ.1.1 contains R346T and K444T substitutions, and BN.1 contains an R346T substitution and a potential *N*-linked glycan at N354. As a result, the COV2-3835 structure highlights the importance of residues K444 and R346 for binding and probably explains why members of this clonotype neutralize XBB.1.5 but not BQ.1.1 or BN.1.

Analysis of the cryo-EM reconstruction of COV2-3891 in complex with the BQ.1.1 S protein revealed a potential basis for broad RBD cross-reactivity to multiple Omicron variants, including those with substitutions within the class 3 antigenic site (Fig. 4f–i and Supplementary Table 5). The HCDR2 and LDCR3 of COV2-3891 both contact the 500s loop of the RBD and together form an extensive network of hydrogen bonds centred around RBD residues 499–503 (Fig. 4g). Many of these hydrogen bonds are contributed by main-chain amide or carbonyl groups, and thus, are likely to be relatively insensitive to substitutions at these positions. The HCDR2 of COV2-3891 also forms hydrogen bonds with the main-chain carbonyl of V445 and the side chain of residue R498, which is conserved across Omicron variants so far (Fig. 4h). Finally, the HCDR3 of COV2-3891 forms interactions with K440 through the side chain of E100a and main-chain carbonyl of W99, with K98 of the HCDR3 stabilizing the loop through interactions with E100a (Fig. 4i). Together, the combination of extensive main-chain contacts and the interactions with substitutions present in Omicron variants (K440 and R498) provides an explanation for how COV2-3891 maintains binding and neutralization activities to both XBB and BQ.1.1.

The crystal structure of COV2-3906 with the XBB.1.5 RBD sheds light on how it recognizes the relatively conserved class 4 site (Fig. 5a,b and Supplementary Table 6). All six CDRs make contacts with RBD residues (Fig. 5c). All LCDRs are involved in making contacts with the 370 s loop and β-sheet of the RBD, including hydrogen bonds with main-chain carbonyl and amide groups (Fig. 5d). In addition to these main-chain contacts, R94 (Kabat numbering) interacts with the main-chain carbonyl and side chain of N370. The hydroxyl group of RBD residue Y369 interacts with Y50 and H58 of the HCDR2, as well as Y91 of the LCDR3 of COV2-3906. A29 and D51 from the LCDR1 and LCDR2 of COV2-3906, respectively, form hydrogen bonds with K378 (Fig. 5e). In the 380s loop of the RBD, S383 and T385 interact with E50 of the LCDR2 of COV2-3906, while Y100a of the HCDR3 forms a hydrogen bond with the hydroxyl group of the T385 side chain. Finally, the HCDR3 of COV2-3906 makes multiple van der Waals contacts with the RBD.

**Fig. 6:**
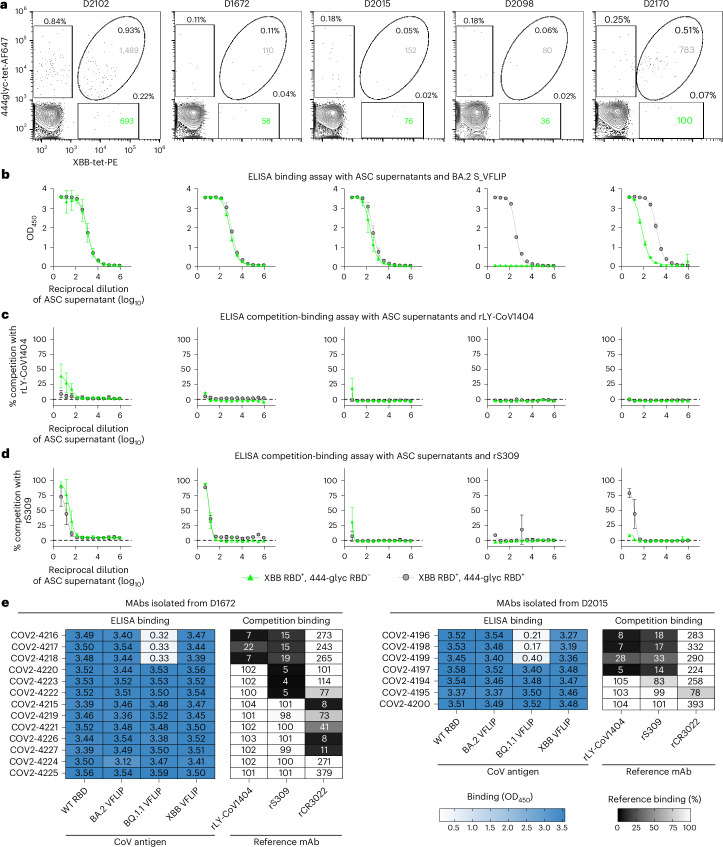
B cell and mAb profiling from additional research participants. **a**, Flow-cytometric enrichment strategy for the isolation of XBB-RBD^+^, 444glyc RBD^−^ B cells as described in Fig. 1. In addition to repeated experiments with D2102, 4 additional participants were profiled (D1672, D2015, D2098 and D2170) to determine the relative B cell frequency of each participant and further validate the enrichment strategy. From each participant, XBB-RBD^+^, 444glyc RBD^−^ (green) and XBB-RBD^+^, 444glyc RBD^+^ (grey) B cells were sorted and expanded on 3T3 feeder layers as previously described. Numbers within each gate indicate the total number of sorted events. **b**, ASC culture supernatants collected from the sorted populations were assessed for binding to the BA.2 S_VFLIP protein by ELISA. No detectable binding was observed for supernatants derived from the XBB-RBD^+^, 444glyc RBD^−^ B cells of D2098, probably because insufficient cells survived and expanded. **c**,**d**, ASC culture supernatants collected from the sorted populations were assessed for the ability to compete with the biotinylated class 3 site-specific reference mAbs rLY-CoV1404 (**c**) or rS309 (**d**) for binding to the BA.2 S_VFLIP protein via a competition-binding ELISA. Reference mAb binding was normalized to binding in the absence of competing supernatant. Competitive binding in the presence of supernatant from XBB-RBD^+^, 444glyc-RBD^−^ cells indicates successful enrichment for B cells targeting the class 3 site. XBB-RBD^+^, 444glyc RBD^−^ ASC supernatants from D2012, D1672 and D2015 showed some level of competition with rLY-COV4104 at the highest concentrations tested. For several participants, the ASC supernatants derived from both sorted populations competed with rS309 for binding in ELISA. For binding and competition-binding curves (**b**–**d**), points and error bars shown are the mean ± s.d. of ELISA signal from serial dilutions of ASC supernatants taken from wells in technical duplicates from an experiment performed once. **e**, ELISA and competition-binding data of mAbs isolated from XBB-RBD^+^, 444glyc RBD^−^ B cells derived from D1672 (left) and D2015 (right). Values for OD_450_ (left) or per cent reference binding (right) are shown for each mAb. As described in Fig. 2, the darker blue colour indicates higher OD_450_ for ELISA binding. The corresponding heat map for competition binding indicates the extent of competition, with black representing 100% competition with the reference mAb and white representing 0% competition with the reference mAb. Similar patterns of competition were observed for mAbs isolated from D1672 and D2015 as for those isolated from D2102 (Figs. 2f and 3), indicating that the enrichment strategy performed similarly for these participants. Reference mAb binding was normalized to binding in the absence of supernatant. Source data

While we succeeded in using this glycan-masking strategy to isolate mAbs recognizing the class 3 antigenic site, our large-scale mAb discovery efforts focused on B cells isolated from a single participant. As a result, it was unclear how common class-3-specific B cells with reactivity to antigenically advanced SARS-CoV-2 variants are across individuals (Fig. 6). To address this question, we used the same flow cytometric sorting strategy to isolate class-3-specific B cells from 5 additional participants, including the previously studied D2102 for direct comparisons. The overall antigen-specific B cell frequency was higher for D2102 relative to the other participants, but a discernible XBB^−^RBD^+^, 444glyc^−^RBD^−^ population was observed for all 5 participants (Fig. 6a). We sorted XBB^−^RBD^+^, 444glyc^−^RBD^−^ and XBB^−^RBD^+^, 444glyc^−^RBD^+^ B cells from each participant. After expanding on 3T3 feeder layers, we again tested ASC culture supernatants for binding to the BA.2 S_VFLIP protein (Fig. 6b) and assessed the ability for antibodies present in the supernatant to bind in the presence of rLY-CoV1404 or rS309. From D2102, we again observed competition with rLY-CoV1404 from the XBB^−^RBD^+^, 444glyc^−^RBD^−^ population, and slight competition from participants D1672 and D2015 (Fig. 6c,d). We obtained paired heavy- and light-chain variable gene sequences from D1672 and D2015, from which we selected and expressed 20 mAbs for binding and competitive-binding analyses. These 20 mAbs were able to bind Wuhan-1 (WT) RBD, and BA.2, BQ.1.1 or XBB_VFLIP trimeric S proteins, confirming the B cell reactivity observed through flow cytometry. Several of these mAbs (COV2-4216 to -4218 and COV2-4196 to -4199 isolated from D1672 and D2015, respectively) competitively blocked rLY-CoV1404 binding (Fig. 6e and Supplementary Table 7). Altogether, these data support the ability to isolate class-3-specific B cells using glycan masking as an enrichment strategy across multiple participants with varying B cell frequencies.

## Discussion

The rapid emergence of SARS-CoV-2 antigenic variants means additional approaches are needed to isolate and characterize mAbs that bind conserved epitopes with desirable antiviral properties. Rationally engineering antigen glycosylation to modulate immune responses is a promising approach in next-generation vaccine design^21–23^. The large size and flexibility of glycans enable shielding of large areas from immune recognition, considerably impacting antigenicity^32^.

Glycan masking has previously been employed to isolate naïve B cells that bind the receptor-binding motif of the SARS-CoV-2 RBD^33^. Here we employed a similar glycan-masking approach to enrich for cross-reactive mAbs that target the class 3 site. Of the panel of isolated mAbs, 31% competed with the receptor blocking class 3 mAb LY-CoV1404 and 61% competed with S309, another class 3 mAb that does not block ACE2 receptor binding. Our structural analyses confirmed the isolation of a diverse set of mAbs with different binding angles targeting the class 3 site and defined how COV2-3891 maintained binding and neutralization to both XBB and BQ.1.1. Although this individual (D2102) had not been exposed to XBB or BQ.1.1 viruses or vaccines, we used our enrichment strategy to isolate rare mAbs that bound to the class 3 site, potently neutralized each of the variants tested, and protected in an animal model of XBB.1.5 infection. Further validating this strategy, we also succeeded in isolating mAbs targeting the class 3 site from additional participants using the same workflow.

Our work provides a proof-of-concept that this glycan-masking approach could be employed to rapidly isolate cross-reactive antibodies that bind epitopes within a given antigenic site. Despite the success of our approach here, analysis of mAb reactivity and epitope specificity revealed areas where our glycan-mask-based enrichment strategy could be improved. Our attempts to express XBB and BQ.1.1 RBDs with a glycan at position 444 were unsuccessful, suggesting that neighbouring residues may influence whether a glycan at this position is tolerated. Some mAbs unexpectedly competed only with the class 4 mAb rCR3022, possibly due to the introduction of the 444-glycan altering the accessibility of the class 4 site in the context of antigen tetramers. This finding suggests that while glycan masking is a useful strategy for performing antigenic site-focused enrichment, glycan engineering may also result in unexpected enrichment of B cells with unintended specificities.

In theory, the glycan-masking approach is not limited to targeting a single site. For example, in future work, multiple glycan-masked antigens could be used in high-throughput barcoding approaches such as LIBRA-seq^34,35^ to rapidly and simultaneously map multiple antibodies to different antigenic sites. As SARS-CoV-2 variants with multiple constellations of antigenic mutations continue to emerge after undergoing prolonged evolution, glycan-mask-based antigen engineering offers a focused and potentially more efficient approach to isolating antibodies with desired reactivity features. Sorting with glycan-masked antigens could also serve as a strategy to rapidly identify highly cross-reactive mAbs that target these epitopes. Data from studies using site-specific masked antigens can also inform rational design of new candidate vaccine antigens by steering the humoral immune response towards epitopes associated with potent neutralization.

## Methods

### Research participants

The Institutional Review Board of Vanderbilt University Medical Center approved human studies (protocol number 8675). All participants gave written informed consent before study participation. Participants were offered compensation for study participation. All ethics regulations for human participant studies were followed. Demographic data and exposure histories for the participants profiled in this manuscript are available in Extended Data Table 2. D2102 was a 28-year-old otherwise healthy female in the United States with a previous history of three mRNA vaccinations encoding the ancestral SARS-CoV-2 sequence (Wuhan-Hu-1). This individual became infected in December 2021 with SARS-CoV-2 at a time when BA.1 and BA.1.1 variants dominated local circulation. Five days after exposure, the individual had upper respiratory illness symptoms and a positive PCR test on nasal secretions for the presence of SARS-CoV-2, suffered a mild clinical illness that resolved on the sixth day, and fully recovered with only supportive therapy. Two months later, a sample of peripheral blood was obtained by phlebotomy after written informed consent and processed for PBMC isolation. This individual subsequently underwent leukapheresis, and PBMCs were purified using a negative-selection-based magnetic enrichment kit (StemCELL Technologies, 19654). After isolation, PBMCs were cryopreserved and stored in a liquid nitrogen freezer until use. For participants D1672, D2015, D2098 and D2170, PBMCs were isolated from peripheral blood draws using the same kit.

### Cell lines

Expi293F cells (Thermo Fisher, A1452) were propagated in Expi293F Expression Medium (Thermo Fisher, A1435102) at 37 °C and 8% CO_2_. A previously described engineered NIH3T3 fibroblast line (originating from a male mouse) constitutively expressing cell-surface human CD40L, BAFF and IL-21 (ref. ^24^) was kindly provided by D. Bhattacharya (University of Arizona, Tuscon, AZ). HEK-293T/17 cells were obtained from The American Type Culture Collection (ATCC, CRL-11268). HEK-293T cells stably transduced to express human ACE2 (293T-hACE2 cells) or 293T cells stably expressing both hACE2 and TMPRSS2 (293T-hACE2-TMPRSS2) were obtained from BEI Resources (NR-52511, NR-55293). Vero-TMPRSS2 cells^36^ were maintained at 37 °C in 5% CO_2_ in 1× Dulbecco’s modified Eagle medium (DMEM, Gibco, 11995-040) supplemented with fetal bovine serum (FBS, 10% v/v), HEPES (10 mM), sodium pyruvate (1 mM), non-essential amino acids, blasticidin (5 µg ml^−1^) and penicillin–streptomycin (100 U ml^−1^). A PCR-based mycoplasma detection kit (ATCC, 30–1012K) was used to test cell cultures on a monthly basis and all tests were negative during the time of study.

### Viruses

The rVSV-SARS-CoV virus was a generous gift from S. Whelan (Washington University, St Louis). The WA1/2020 recombinant strain containing the D614G substitution was described previously^37,38^. The SARS-CoV-2 variant isolates XBB.1.5, EG.5.1 and BQ.1.1 were generous gifts from Y. Kawaoka (University of Wisconsin-Madison), M. Suthar (Emory University) and A. Pekosz (Johns Hopkins University), respectively. All authentic SARS-CoV-2 isolates were expanded once on Vero-TMPRSS2 cells and next-generation sequencing^39^ was used to confirm expected sequences. All experiments involving authentic SARS-CoV-2 were approved by the institutional biosafety committees of either Washington University, St Louis (protocol number 14366) or UNC-Chapel Hill (protocol numbers 202517662, 202517170). Studies were conducted in certified biosafety level 3 (BSL-3) facilities using positive-pressure respirators.

### Recombinant antigen expression and purification

To express the RBD portion of the SARS-CoV-2 S protein, a synthesized cDNA encoding residues 328–531 was inserted into a mammalian expression vector downstream of a phosphatase-mu signal peptide and including C-terminal AviTag and 8×His tags (COV2-RBD_His). Some constructs contained an additional FLAG or myc tag, although these were not used and did not affect protein expression or purification. Three previously identified stabilizing mutations (Y365F, F392W, V395I)^40^ were introduced to improve yield and protein stability. Mutations corresponding to each Omicron subvariant were introduced in the context of these three stabilizing mutations. Expi293F cells were transfected with plasmids encoding each RBD, and expressed proteins were purified by affinity chromatography with HisTrap Excel or TALON HP columns (Cytiva). For crystallographic and binding studies, we also expressed RBD constructs containing an IL-2 signal peptide (MYRMQLLSCIALSLALVTNS), residues 319–528 of the RBD, a Prescission 3C protease cleavage site, an AviTag, a tobacco etch virus (TEV) protease cleavage site, a TwinStrep tag and an 8×His tag (COV2-RBD_Strep). We also introduced Y365F, F392W and V395 mutations to enhance stability and yield. We expressed these COV2-RBD_Strep constructs in Expi293F cells, purified using StrepTrap XT columns (Cytiva) and eluted with 100 mM biotin in 100 mM Tris-HCl pH 8 + 150 mM NaCl. Recombinant protein size, glycosylation state and purity were assessed by SDS–PAGE. For BA.1 constructs where the 444 glycan was introduced, site-directed mutagenesis was used to introduce K444N and S446T mutations, on the basis of previous observations that sequons containing threonine promote more efficient glycosylation than those containing serine^41^.

To express SARS-CoV-2 S proteins for ELISA binding and electron microscopy (EM) studies, we introduced the mutations of the BA.2, XBB and BQ.1.1 variants into the background of a previously described stabilized S protein construct (VFLIP) that was originally based on the Wuhan-Hu-1 sequence^42^. This construct contains a C-terminal T4 fibritin foldon domain, an 8×His tag and a TwinStrep tag. For expression and purification of recombinant soluble SARS-CoV S protein, we used the previously described 2P prefusion-stabilizing substitutions, and this construct also contained a C-terminal T4 fibritin foldon domain, an 8×His tag and a TwinStrep tag^43^. To express proteins, plasmids encoding each prefusion-stabilized S protein construct were transfected into Expi293F cells (Thermo Fisher), and at 4–5 days post transfection, culture supernatants were clarified by centrifugation and sterile filtered using a 0.2 µm filter after the addition of BioLock (IBA Biosciences). Recombinant S protein ectodomains were purified by streptactin-based affinity chromatography using StrepTrap HP or StrepTrap XT columns (Cytiva) and eluted with 25 mM desthiobiotin in 1× Dulbecco’s phosphate buffered saline (DPBS) (for StrepTrap HP) or 100 mM biotin in 100 mM Tris-HCl pH 8 + 150 mM NaCl (for StrepTrap XT). For COV2-3835 structural studies, SARS-CoV-2 Omicron BA.1 mutations were expressed in the background of Hexapro (6P), a trimer construct containing 6 previously described stabilizing mutations, and purified as previously described^44^. Sequences for antigen constructs used for binding assays, antigen-specific sorting and structural studies are included in Supplementary Table 8.

### Modelling of native and engineered SARS-CoV-2 RBD glycans

We used a glycan modelling tool to visualize the location and relative size of RBD glycans^45^. To design the construct, we uploaded a crystal structure of the RBD (PDB: 6M0J) to the GlycoSHIELD web application (http://www.glycoshield.eu/). We selected a high-mannose *N*-linked glycan (Man9) and used the GlycoSHIELD interface to simulate conformations of the glycans and graft each glycan at positions 343 (the site of a native *N*-linked glycan in RBD) and 444 (the location of our engineered glycan mask). We used default grafting settings with 30 glycan conformers for display on the structure. Structures were then visualized using PyMOL v.2.5.7 (Schrödinger).

### Biotinylation of SARS-CoV-2 RBD antigens and tetramer preparation

Site-specific biotinylation of COV2-RBD_His antigens was performed through labelling of the AviTag amino acid sequence with biotin by BirA according to manufacturer protocol (Avidity, BirA500). Briefly, 40 μM of AviTag-containing SARS-CoV-2 RBD, 2.5 μg of BirA biotin-protein ligase, 1× BiomixA and 1× BiomixB were mixed and incubated at room temperature for 1 h to ensure rapid biotinylation. Biotinylation of RBDs was confirmed by ELISA using avidin conjugated to horseradish peroxidase (HRP) (Thermo Fisher, 434423) and TMB substrate (Thermo Fisher, 34029). Colorimetric signal was monitored and the reaction was stopped with 1 M hydrochloric acid. Absorbance then was measured at 450 nm using a plate reader (Biotek). For preparation of tetramers, biotinylated SARS-CoV-2 RBDs were incubated with fluorophore-conjugated streptavidin. Briefly, streptavidin-PE (Thermo Fisher, SA10041) was added to biotinylated XBB or BQ.1.1 RBDs, and streptavidin-Alexa Fluor 647 (AF647; Thermo Fisher, S21374) was added to biotinylated BA.1-444glyc RBD at a molar ratio of 4:1. Fluorophore-conjugated streptavidin was added stepwise over the course of 10 different steps, with a brief mix in between steps to maximize tetramer formation. Each addition of streptavidin to the RBDs was followed by incubation at room temperature for 10 min. Reactions were then incubated overnight at 4 °C to complete tetramer formation.

### B cell enrichment and flow cytometric cell sorting

Cryopreserved PBMCs (1 × 10^8^ total cells) were thawed in a 37 °C water bath and immediately mixed with cold RoboSep buffer (StemCELL Technologies, 20104). After a brief centrifugation (250 × *g*, 5 min) at room temperature, the cell pellet was resuspended in cold RoboSep buffer. B cells were enriched using a negative-selection magnetic bead-based enrichment kit (EasySep Human B Cell Isolation kit, StemCELL Technologies, 17954) according to manufacturer protocol. After washing with cold RoboSep buffer (250 × *g*, 5 min) at room temperature, the isolated B cells were incubated with a cocktail of phenotyping antibodies, each at a 1:20 dilution, for 45 min on ice. The phenotyping antibodies included: anti-human CD19–Brilliant Violet 421 (BioLegend, clone HIB19, 302234), anti-human IgD–FITC (BioLegend, clone IA6–2, 348206) and anti-human IgM–FITC (BioLegend, clone MHM-88, 314506). The isolated B cells were then centrifuged briefly, washed with RoboSep buffer (250 × *g*, 3 min) and incubated with SARS-CoV-2 RBD tetramers, each at a final concentration of 1 µg ml^−1^, on ice for 45 min. Two separate reactions were performed using either the XBB or BQ.1.1 RBD tetramers-PE and BA.1-444glyc RBD tetramers-AF647. Cells were then washed briefly with RoboSep buffer (250 × *g*, 3 min) and resuspended in 500 µl of RoboSep buffer for flow cytometric analysis using an SH800 cell sorter (Sony Biotechnology). Flow cytometric data were analysed with the SH800 software (Sony Biotechnology) and FlowJo v.10 (Tree Star). XBB or BQ.1.1 RBD-reactive and BA.1-444-glyc RBD-non-reactive B cells (1,345 XBB- and 462 BQ.1.1-reactive) were sorted into Medium A (StemCELL Technologies, 03801, 1000041736) containing penicillin and streptomycin. Cells were then expanded for 8 days in the presence of CpG and irradiated 3T3 feeder cells expressing human CD40L, IL-21 and BAFF, as previously described^24^. Expanded ASCs were screened and confirmed by ELISA for secretion of SARS-CoV-2 RBD-specific antibodies. Expanded ASCs were separated from irradiated 3T3 feeder cells through flow cytometric cell sorting. For antibody sequence recovery from participant D2102, 40,000 expanded ASCs were stained with anti-human CD45-PE (BioLegend, 368509). After, flow cytometric sorting, ~25,000 cells were prepared for loading onto the OptoSelect 11k chip of a Beacon optofluidic system (Bruker Cellular Analysis, formerly Berkeley Lights) for single-cell analysis. The remaining cells were sequenced using the 10x Genomics Chromium sequencing method to generate paired antibody–variable gene libraries. For antibody sequence recovery from participants D1672 and D2105, ASCs were sorted into 96-well PCR plates containing lysis buffer.

### Single-cell optofluidic assay selection of SARS-CoV-2 RBD-reactive B cells

Expanded ASCs were screened using a Beacon optofluidic system (Bruker Cellular Analysis). First, cells were loaded onto OptoSelect 11k chips in a plasmablast survival medium, which promotes antibody secretion and maintains cell viability^46^. Next, thousands of ASCs were transferred into individual nl-volume chambers (NanoPens) across the chip using opto-electropositioning (OEP). To screen ASCs for binding reactivity, 6–8-µm (BLI assay beads, 520-00053) and 10–14-µm (Spherotech, SVP-100-4) beads were prepared by coupling biotinylated XBB or BQ.1.1 RBDs to streptavidin-coated polystyrene particles. Conjugated beads were prepared at final concentrations of 5% (w/v) for 6–8 μm beads and 0.5% (w/v) for 10–14 μm beads. These conjugated beads were mixed with AF568-labelled anti-human IgG (H + L) cross-adsorbed secondary antibodies (Thermo Fisher, A-21090) at a 1:2,500 dilution for detection of secreted RBD-reactive antibodies. This mixture was imported onto the OptoSelect 11k chips for an on-chip, fluorescence-based assay. In this assay, positive SARS-CoV-2 RBD binding reactivity was detected through antibody binding to the conjugated beads and sequestration of fluorescent signal (AF568) from the secondary antibodies. Fluorescent signal on beads adjacent to individual NanoPens was used to identify B cells secreting XBB or BQ.1.1 RBD-reactive antibodies. An on-chip in-pen assay was also performed to select for antibodies that blocked hACE2 binding to either XBB or BQ.1.1 RBDs. In this surrogate hACE2 blocking assay, 10–14-µm XBB or BQ.1.1 RBD-conjugated streptavidin-coated beads (Spherotech) were loaded into NanoPens containing individual ASCs and incubated to allow saturation of the RBD with secreted antibodies. Then, a mixture containing recombinant hACE2 with a FLAG tag (10 µg ml^−1^, Sigma-Aldrich, SAE0064), a rat anti-FLAG tag AF647 antibody (1:100 dilution, BioLegend, clone L5, 637315, B265929) and anti-human IgG (H + L) cross-adsorbed AF568 antibodies (1:200 dilution, Thermo Fisher, A-21090) was perfused throughout the OptoSelect 11k chip for diffusion into the NanoPen chambers. Cells secreting antigen-reactive antibodies were identified by fluorescent signal (AF568) from XBB or BQ.1.1 RBD-conjugated beads using the Beacon TRED filter cube. Simultaneously, AF647 signal was detected using a Cy5 filter cube as a measure of hACE2 binding. NanoPen chambers that contained fluorescent XBB or BQ.1.1 RBD-conjugated beads in both fluorescence channels were considered to contain B cells secreting XBB or BQ.1.1 RBD-reactive antibodies that were unable to block hACE2 binding at the concentrations tested. In contrast, NanoPens that contained fluorescent XBB or BQ.1.1 RBD-conjugated beads in the TRED channel but not in the Cy5 channel were classified to contain B cells secreting XBB-RBD-reactive and hACE2-blocking antibodies. Cells with activities of interest were exported from specific NanoPens by OEP into lysis buffer in individual wells of 96-well RT–PCR plates for antibody sequencing.

### Sequencing of B cells following optofluidic functional screening or single-cell sorting

After export of D2102 ASCs that bound to XBB or BQ.1.1 from the Beacon instrument, components of the Opto Plasma B Discovery cDNA Synthesis kit (Berkeley Lights, 750-02030) were used to amplify and recover antibody heavy- and light-chain sequences. The Opto Plasma B Discovery Sanger Prep kit (Berkeley Lights, 750-02041) was used to perform further rounds of sequence amplification, and amplicons were sequenced using Sanger sequencing (Azenta Life Science). This same amplification and sequencing workflow was used to recover the heavy- and light-chain sequences of ASCs from D1672 and D2015. Sequence data were analysed using a Python-based antibody variable-gene analysis tool (PyIR; https://github.com/crowelab/PyIR)^47^ to control the quality of the sequences selected. Subsequent steps were performed only for those clones for which full-length, unambiguous, paired heavy- and light-chain variable-gene sequences were obtained.

### Chromium sequencing of single B cells through 10x Genomics

Expanded ASC populations derived from XBB- or BQ.1.1-binding B cells were sequenced using the Chromium single-cell sequencing platform following vendor protocol (10x Genomics). Briefly, ~40,000 XBB-reactive or 20,000 BQ.1.1-reactive ASCs were split evenly into replicates of 10,000 cells each and separately added to a reverse-transcription reaction mix (following vendor protocol), which was then loaded directly onto a Chromium chip for cell and barcoded bead encapsulation (10x Genomics, PN-1000263). Following reverse transcription, sequencing libraries were prepared following the User Guide for Chromium NextGEM Single Cell 5′ Reagent Kits v2 (CG000331 Rev C). Briefly, total cDNA was amplified, after which BCR sequences were amplified using the Chromium Single Cell Human BCR Amplification kit (10x Genomics, PN-1000253) according to the User Guide for Chromium Single Cell 5′ Reagent Kits v2 (CG000331 Rev C). The enriched libraries were sequenced using a NovaSeq 6000 S4 Reagent kit (Illumina) on a NovaSeq sequencer for 300 cycles. As suggested by the Chromium User Guide, a sequencing depth of greater than 5,000 raw reads was selected for each sample on the basis of input cells.

### Bioinformatic analysis of antibody–variable gene libraries

Following next-generation sequencing, all samples were demultiplexed and the 10x Genomics Cell Ranger software (v.6.1.2) was used to process FASTQ files. Following the Cell Ranger bioinformatic processing, we collected all the heavy- and light-chain paired sequences for which there was a 1:1 pairing and processed those sequences using PyIR^47^. We next excluded sequences from downstream processing if they: (1) contained a stop codon, (2) did not encode an intact heavy- or light-chain CDR3 and (3) did not contain an in-frame junctional region. In a second phase of processing, we deduplicated sequences to exclude those with identical amino acid sequences. Paired antibody sequences that were assigned as IgM on the basis of Cell Ranger analysis were not considered. PyIR was used to determine germline gene usage, define CDRs and identify somatic mutations relative to germline sequences for each paired antibody sequence.

### Bioinformatic downselection of antibody panel using paired sequence clustering

Paired, antigen-specific antibody sequences were clustered on the basis of genetic similarity to identify clonal families. Sequences were first binned together if they were encoded by the same inferred heavy-chain *V* and *J* gene and had the same HCDR3 length. Next, sequences were clustered according to 80% identity on the HCDR3 nucleotide sequence using the single-linkage clustering algorithm^48^, implemented by SciPy^49^. Then, the antibody sequences were grouped together on the basis of shared *V* and *J* gene and LCDR3 length. Finally, sequences were again clustered according to 80% identity on the LCDR3 nucleotide sequence using the single-linkage algorithm. We defined these clusters of sequences as clonal families. The most somatically mutated sequence from each clonal family, as determined by PyIR, was selected for synthesis and expression. If there were multiple members of a clonal family, the sequence closest to the consensus sequence was also selected for synthesis and expression.

### Microscale and ‘midi-scale’ expression of recombinant mAbs and Fabs

To express a large panel of ~300 mAbs, a microscale expression platform was used. Briefly, ~1 ml per well of ExpiCHO cell cultures was transfected using the Gibco ExpiCHO expression system in deep 96-well plates (Thermo Fisher), as previously described^25,26^. For high-throughput microscale mAb purification, supernatants were clarified by centrifugation and incubated with MabSelect SuRe resin (Cytiva). The resin was then washed with 1× DPBS and filtered deionized water, followed by elution and neutralization with 0.1 M sodium acetate pH 3.3 buffer and 5 M Tris-HCl pH 8.0, respectively. Eluted mAbs were then buffer exchanged into 1× DPBS using Zeba spin desalting plates (Thermo Fisher) and stored at 4 °C until functional analyses were performed. To generate larger amounts of recombinant mAbs, ‘midi-scale’ expressions were performed. In this case, ~15–30 ml ExpiCHO cell cultures were transfected using the Gibco ExpiCHO expression system as described by vendor protocol. For high-throughput ‘midi-scale’ mAb purification, mAbs were purified from clarified cell culture supernatants using HiTrap MabSelect SuRe (Cytiva) columns on a 24-column parallel protein chromatography system (Protein BioSolutions). Following purification, mAbs were buffer exchanged into 1× DPBS, concentrated using Amicon Ultra 50-kDa-cutoff centrifugal filter units (Millipore Sigma) and stored at 4 °C until use. For recombinant Fab production for high-resolution structural studies, constructs encoding the heavy chain variable region and CH1 domain, and the light chain variable and constant regions were transfected into ExpiCHO cells using the same procedure as for recombinant mAb production. Recombinant Fabs were purified from culture supernatant using an anti-CH1 CaptureSelect column (Thermo Fisher).

### High-throughput mAb quantification

High-throughput quantification of recombinantly expressed mAbs was performed using purified mAbs in a 96-well plate. The Cy-Clone plus kit was used according to vendor protocol and analysis performed on an iQue Plus Screener flow cytometer (Sartorius). Purified mAbs were measured at a single dilution (final dilution of 1:10, 2 μl of mAb per reaction). The concentration of mAb was interpolated on the basis of the relative competition signal relative to a standard curve of a control human IgG. Data were analysed using the ForeCyt software v.6.2 (Sartorius).

### ELISA dose–response binding assays

Microtitre plates (384-well) were coated with purified recombinant SARS-CoV-2 RBD proteins (diluted 2 µg ml^−1^ in 1× DPBS) in a volume of 25 µl well^−1^ at 4 °C overnight. The next day, plates were washed with 1× DPBS containing 0.05% Tween-20 (DPBS-T) and blocked with blocking buffer (2% (w/v) non-fat dry milk and 2% (v/v) normal goat serum in DPBS-T) for 1 h at room temperature. Each mAb was diluted in blocking buffer at a starting concentration of 10 µg ml^−1^ and serially diluted 3-fold for a 12-point dilution series. Plates were then washed and 25 µl of each mAb dilution were added to each well and incubated for 1 h at room temperature. Plates were washed and 25 µl of blocking buffer and goat anti-human IgG secondary antibody conjugated with HRP (Southern Biotech, 2014-05, L2118-VG00B, 1:5,000 dilution in blocking buffer) were added to each well. Plates were then incubated for 1 h at room temperature. After plates were washed, 25 µl of 3,3′,5,5′-tetramethylbenzidine (TMB) substrate (Thermo Fisher) was added and the plates incubated at room temperature to develop the signal. Following sufficient signal development, the reaction was stopped by addition of 25 µl 1 M hydrochloric acid and absorbance was measured at 450 nm using a spectrophotometer (Biotek). ELISA dose–response binding assays were performed with technical triplicates in at least 2 independent experimental replicates.

### SARS-CoV-2 S-pseudotyped lentivirus generation and titration

Single-cycle lentiviruses pseudotyped with the S glycoproteins of SARS-CoV-2 variants were generated using a previously described protocol^50^. Briefly, HEK-293T/17 cells (ATCC, CRL-11268) were seeded in a T-225 cm^2^ culture flask targeting 50–70% confluency the following day. The morning of the transfection, the medium was changed to fresh DMEM supplemented with sodium pyruvate (Thermo Fisher, 11995073), Ultra-Low IgG FBS (10% v/v, Gibco, 16250078) (DMEM + 10%), HEPES (25 mM, Gibco, 15630-080) and penicillin/streptomycin (Gibco, 15140122). To generate lentiviral-based reporter pseudotyped viruses, we used components of a SARS-CoV-2 lentiviral pseudotyping kit (BEI NR-53817). Briefly, 22.5 µg of the lentiviral genome plasmid pHAGE-CMV-Luc2-IRES-ZsGreen-W (BEI Resources, NR-52516), 4.95 µg each of the packaging plasmids pRC-CMV- Rev (BEI Resources, NR-52519), HDM-Hgpm2 (BEI Resources, NR-52517) and HDM-tat1b (BEI Resources, NR-52518), and 7.65 µg of a CMV-driven plasmid encoding a codon-optimized SARS-CoV-2 S variant gene with a 21-amino-acid deletion were added to 1 ml of serum-free DMEM. After mixing, 45 µl of BioT transfection reagent (Bioland Scientific, B01-00) was added and the plasmid:transfection reagent mixture was mixed gently and incubated at room temperature for 5 min. Following incubation, the transfection mixture was added dropwise to the flask while swirling gently. Approximately 16 to 18 h later, the medium was removed and fresh DMEM supplemented with sodium pyruvate (Thermo Fisher, 11995073), penicillin/streptomycin (Gibco, 15140122), 25 mM HEPES (Gibco, 15630-080) and 2% (v/v) Ultra-Low IgG FBS (Gibco, 16250078) (DMEM + 2%). Supernatants were collected ~48 h after transfection, centrifuged to remove cells and filter sterilized to further remove cellular debris. Single-use aliquots of pseudotyped virus stocks were prepared and stored at −80 °C.

### Lentiviral pseudotyped virus neutralization assays

Lentivirus-based pseudotyped viral neutralization assays were performed according to a previously described protocol^50^. One day before, poly-D-lysine-coated 96-well tissue culture plates (Thermo Fisher, A3890401) were seeded with HEK-293T cells stably expressing human ACE2 (293T-hACE2 cells, BEI Resources NR-52511) at a density of 1.25 × 10^4^ cells per well in DMEM + 10%. The following day, 4-fold serial dilutions of mAbs were prepared in DMEM + 2% in a 96-well polypropylene microtitre plate. mAbs dilutions were then mixed with pseudotyped virus for 1 h at 37 °C. Polybrene (5 µg ml^−1^, EMD Millipore) was present to enhance pseudovirus infection. After the incubation, pseudotyped virus–mAb mixtures were added to 293T-hACE2 cell monolayers. Plates were incubated at 37 °C for 48–60 h, at which point cells were lysed using the Bright-Glo Luciferase Assay System (Promega). Luciferase activity was then quantified using a CLARIOStar plate reader (BMG LabTech). Wells in which neither pseudotyped virus nor mAb was added were used to determine the average background luminescence signal from each well, which was then subtracted from readings. The percent infection of each well was then determined relative to the average signal from wells in which only pseudotyped virus was added. Four-parameter (inhibitor) vs response curves were fit to the data using nonlinear regression in Prism v.9.5 (GraphPad). IC_50_ values were defined by constraining the top or bottom values of curve fits to 100 or 0, respectively. Each neutralization assay was executed in technical duplicate for each mAb and performed in at least two independent experiments. For neutralization assays with KP.3, 293T-hACE2-TMPRSS2 cells were used instead of 293T-hACE2 cells, with no other alterations to the assay protocol.

### rVSV-SARS-CoV virus neutralization assay

To screen for the neutralizing activity of SARS-CoV S (S2P)-reactive mAbs, we used a previously described impedance-based real-time cellular analysis (RTCA) assay and an xCelligence RTCA HT Analyzer (Agilent). RTCA measures changes in electrical impedance that are associated with changes in cell physiology, including the virus-induced cytopathic effect (CPE)^51^. Briefly, 50 µl of cell culture medium (DMEM + 2%) was added to each well of a 96-well plate containing gold electrodes (E-plate, Agilent) and the plate was measured to establish background impedance. Vero CCL81 cells were then added to each well (18,000 cells per well in a volume of 50 μl DMEM + 2%) and the plate was placed back on the analyser. At 17–20 h after seeding, 60 µl of a recombinant VSV expressing the S protein of SARS-CoV (rVSV-SARS-CoV, 5,000 p.f.u.s) was mixed with 60 µl of 3-fold serially diluted antibodies to make a total volume of 120 μl using DMEM + 2% diluent and incubated for 1 h at 37 °C in 5% CO_2_. After incubation, the virus–mAb mixtures were added to 96-well E-plates without removal of culture medium. Wells containing virus only (in the absence of mAb or negative control mAb) and wells containing only Vero CCL81 cells in the medium were included as controls. Plates were measured for 68–72 h to assess virus neutralization. Sensograms were visualized using RTCA HT software v.1.0.1 (ACEA Biosciences). Antibodies were assessed in technical duplicates at a starting concentration of 10 µg ml^−1^. Neutralization was calculated as a percentage of cell index in control wells where no virus was added after subtraction of the cell index of virus-only wells that exhibited maximal CPE ~40–48 h after addition to cells.

### Authentic virus neutralization assays

Serial dilutions of mAbs at a starting concentration of 10 μg ml^−1^ were incubated with 10^2^ focus-forming units (f.f.u.s) of WA1/2020 D614G, XBB.1.5, EG.5.1 or BQ.1.1 for 1 h at 37 °C. Antibody–virus mixtures were added to Vero-TMPRSS2 cells seeded the day before in 96-well plates. Antibody–virus mixtures were incubated with cells at 37 °C for 1 h to allow virus internalization. Next, an overlay of 1% (w/v) methylcellulose in MEM was added to cells. Methylcellulose overlays were removed 30 h (WA1/2020 D614G) or 72 h (XBB.1.5, EG.5.1, BQ.1.1) later and cell monolayers were fixed with paraformaldehyde (4% in PBS, v/v) for 20 min at room temperature (r.t). Plates were then washed and incubated with a pool of anti-S antibodies of mouse origin^52^ (SARS2-02, -08, -09, -10, -11, -13, -14, -17, -20, -26, -27, -28, -31, -38, -41, -42, -44, -49, -57, -2, -64, -65, -67 and -71). This pool included antibodies with cross-reactivity to SARS-CoV. After washing, an HRP-conjugated goat anti-mouse IgG secondary antibody (Sigma, A8924) was added to detect bound anti-S antibody. Staining steps were performed in PBS supplemented with 0.1% saponin and 0.1% bovine serum albumin. After washing, SARS-CoV-2-infected cell foci were detected by addition of TrueBlue peroxidase substrate (KPL). Foci were imaged and quantitated on an ImmunoSpot microanalyzer (Cellular Technologies).

### Competition-binding ELISA

Wells of 384-well microtitre plates were coated with 1 μg ml^−1^ of purified SARS-CoV-2 BA.2 VFLIP ectodomain protein at 4 °C overnight. Plates were blocked with blocking buffer (2% BSA in DPBS-T) for 1 h at r.t. Unlabelled mAbs were diluted 10-fold in blocking buffer and added to wells (20 μl per well). The plates were then incubated for 1 h at room temperature. Biotinylated preparations of recombinantly expressed reference mAbs rLY-CoV1404, rS309 or rCR3022 were then added to each respective mAb at 2.5 μg ml^−1^ in a volume of 5 μl per well (final concentration of 0.5 μg ml^−1^) without previous washing of the unlabelled mAbs. Plates were then incubated for 1 h at r.t. Plates were washed with DPBS-T and incubated with HRP-conjugated avidin (Sigma-Aldrich, A3151) for 1 h at r.t. Bound mAbs then were detected by addition of 25 μl of a TMB substrate and the reaction was stopped by addition of 25 μl 1 M HCl. Background signal was subtracted, and binding signal was normalized to the binding of each biotinylated reference mAb in the absence of competing mAbs. The following criteria were used to determine competition with the reference mAbs: <33% of the maximal binding signal of the reference mAb indicates full competition, 33–67% indicates partial competition, and >67% indicates no competition.

### hACE2 competition-binding ELISA

Assays to measure the ability of mAbs to compete with hACE2 for binding to S were completed as previously described^18^. Briefly, 384-well microtitre ELISA plates were coated with 2 μg ml^−1^ purified recombinant SARS-CoV2-S_VFLIP proteins in a total volume of 25 μl at 4 °C overnight. The following day, plates were washed with DPBS-T and blocked with 2% non-fat dry milk and 2% normal goat serum in DPBS-T (blocking buffer) for 1 h at r.t. After blocking, plates were washed with DPBS-T and 2-fold serial dilutions of mAbs at a starting concentration of 10 μg ml^−1^ were added to the wells (20 μl well^−1^ in blocking buffer) and incubated for 1 h at r.t. Recombinant hACE2 with a C-terminal FLAG tag (Sigma-Aldrich, SAE0064) was added to wells at 2 μg ml^−1^ in a 5 μl volume of blocking buffer (final 0.4 μg ml^−1^ concentration of hACE2 following addition to each well) without washing. Plates were incubated for 40 min at ambient temperature. Plates were then washed with DPBS-T and an HRP-conjugated anti-FLAG antibody (Sigma-Aldrich, A8592, 1:5,000 dilution in blocking buffer) was added to detect bound hACE2. After 1 h incubation at r.t, plates were washed with DPBS-T and signal was developed by addition of 25 µl TMB substrate followed by 25 µl 1 M HCl. ACE2 binding without competing antibody served as a control. The signal obtained for hACE2 binding at each dilution of tested antibody was expressed as a percentage of the hACE2 binding signal in wells without antibody. IC_50_ values for mAb inhibition of hACE2 binding to COV2-S_VFLIP proteins were determined after log transformation of antibody concentration using sigmoidal dose–response nonlinear regression analysis (Prism software, GraphPad Prism v.8.0). Each mAb dilution series was performed in triplicate except for the SA55 positive control mAb, which was performed in duplicate. Each assay was repeated in 2 independent experiments.

### MAb passive-transfer protection studies in mice

Animal studies were carried out in accordance with the Institutional Animal Care and Use Committee at UNC-Chapel Hill (protocol no. 23-085). The hACE2-K18 mice used for this study were bred at UNC and original breeding pairs were obtained from the Jackson Laboratory (034860). Twelve-month-old hACE2-K18 mice of both sexes were treated with 200 μg of mAb or isotype-matched control mAb via intraperitoneal injection 24 h before viral challenge. Mice were anaesthetized with a mixture of ketamine/xylazine, inoculated intranasally with 10^4^ p.f.u.s of SARS-CoV-2 (XBB.1.5) and monitored daily for clinical signs of disease, weight loss and mortality. At the indicated times after infection, mice were euthanized via isoflurane overdose, and the inferior lung lobe was collected in PBS with glass beads and stored at −80 °C for viral titre determination via plaque assay as previously described^53^. Briefly, lung homogenates were serially diluted in PBS and 200 µl of diluted homogenate were added to Vero E6 cells, followed by the addition of an agarose overlay. Three days following infection, wells were stained with neutral red and plaques were quantified.

### Negative-stain electron microscopy sample and grid preparation, imaging and processing of S-Fab complexes

To perform negative-stain electron microscopy (nsEM), Fab molecules were produced by digesting purified IgG molecules using a resin-immobilized cysteine protease enzyme (Genovis, A2-AFK-100). The digestion occurred in 100 mM sodium phosphate, 150 mM NaCl pH 7.2 (PBS) for ~16 h at ambient temperature. Following digestion, the reaction mix was incubated with CaptureSelect IgG-Fc (multispecies) resin (Thermo Fisher) for 30 min at ambient temperature in PBS buffer to remove intact IgG and cleaved Fc.

For screening and data collection of XBB or BQ.1.1 VFLIP S proteins in complex with Fab molecules, the proteins were incubated at a molar ratio of 4:1 (Fab:S) for ~1 h at r.t. and ~3 µl of the sample at concentrations of ~10–15 µg ml^−1^ was applied to a glow-discharged grid with continuous carbon film on 400-square-mesh copper EM grids (Electron Microscopy Sciences). Uranyl formate (2%) was used for staining^54^. Images were collected using an FEI TF20 (TFS) transmission electron microscope equipped with a Gatan US4000 4k × 4k CCD camera operated at 200 keV and controlled with SerialEM^55^. All imaging was performed at ×50,000 magnification, resulting in a pixel size of 2.18 Å pixel^−1^ in low-dose mode at a defocus of 1.5–1.8 μm. The total dose for the micrographs was ~30 e^−^ Å^−2^. The cryoSPARC software package was used for image processing^56^. Images were imported, and micrographs were contrast transfer function (CTF)-estimated and particles autopicked. The particles were extracted with a box size of 256 pixels and binned by 2–128 pixels (4.36 Å pixel^−1^). Class averages (2D) were performed and good classes were selected for ab initio model and non-uniform (NU) refinement with or without symmetry depending on the occupancy of the Fab molecules. The final resolution of the maps was ~20–26 Å. ChimeraX^57^ was used for model docking, segmentation of the nsEM map and figure preparation. PDB IDs 7LRT and 12E8 were used for the S trimer protein and Fab molecules, respectively. All nsEM data have been deposited in the EMDB with accession codes EMD-43882 through EMD-43888.

### Cryo-EM sample and grid preparation for the BA.1 spike protein in complex with COV2-3835 Fab molecules

The BA.1 spike protein was mixed to a final concentration of 0.65 mg ml^−1^ with a 1.25× molar excess of COV2-3835 Fab molecules in buffer containing 2 mM Tris pH 7.5, 200 mM NaCl and 0.02% NaN_3_. Of the complex, 4 μl was applied to UltrAufoil R 1.2/1.3 300-mesh gold TEM grids (Electron Microscopy Sciences) that had been glow discharged using a PELCO easiGlow (Ted Pella) at a current of 20 mA for a total of 60 s. Using a Vitrobot Mark IV (Thermo Fisher), a blot force of 2 was applied for 3 s to blot away excess liquid before plunge freezing into liquid ethane. Samples were blotted in 100% humidity at 22 °C. A total of 3,096 videos were collected from a single grid using a Titan Krios G3 TEM (Thermo Fisher) equipped with a Biocontinuum Imaging Filter (Gatan), set to a slit width of 20 eV and a K3 direct electron detector (Gatan). SerialEM v.4.0.10 software was used for automatic collection of all videos^55^. Particles were imaged with an exposure of 14 eps for 3.8 s (total exposure of 60 e Å^−2^) at a calibrated magnification of 0.84 Å pixel^−1^. Additional details about data collection can be found in Extended Data Fig. 6 and Supplementary Table 3.

### Cryo-EM data processing and structure building for the BA.1:COV2-3835 Fab complexes

Motion correction, CTF estimation, particle picking and preliminary two-dimensional (2D) classification were performed using cryoSPARC v.4.1.1 live processing^56^ (Extended Data Fig. 6 and Supplementary Table 3). After data collection was completed, 1,127,216 extracted particle picks (box size: 512 pixels, Fourier cropped to 160 pixels) were sorted into 100 2D class averages using an uncertainty factor of 1 to determine and eliminate ‘junk’ classes. From this, 522,475 particles from 25 classes were selected and a second round of 2D classification was performed using an uncertainty factor of 2 to increase diversity of higher-quality classes. A total of 498,988 particles were selected, and 200,000 were used to perform a 4-class ab initio reconstruction. All particles were subsequently used to perform a heterogeneous refinement of the 4 ab initio volumes. Particles from the 2 highest-quality classes were selected and another ab initio reconstruction was performed using 5 classes to aid in sorting out any remaining ‘junk’ particles. From the subsequent refinement of these volumes, particles from the 2 highest-quality classes were further sorted into 3 classes using 3D classification limited to 8 Å resolution to observe subtle heterogeneity in the particles due to flexibility and/or mixed Fab occupancy. A total of 153,719 particles from a single 3D class were refined using homogeneous refinement with no applied symmetry, followed by non-uniform refinement. After re-extracting the particles without binning, an additional non-uniform refinement was performed with no applied symmetry, and with refined per-particle defocus and per-group CTF parameters^58^. The resulting map reached a global resolution of 2.8 Å, although resolution of the RBD–Fab interface was limited due to RBD flexibility. The refined volume was imported into ChimeraX^59^ to generate a mask encompassing a single Fab molecule bound to the RBD and most of the N-terminal domain (NTD) of the neighbouring protomer. The mask was then imported back into cryoSPARC to perform focused refinement of the final global reconstruction to yield a 3 Å reconstruction of the RBD bound to COV2-3835 Fab molecules. To improve map quality, the focused refinement volume was processed using DeepEMhancer within COSMIC^2^ science gateway^60^. An initial model of the complex was generated by fitting the RBD (residues 329–529) from a high-resolution model of the SARS-CoV-2 Omicron BA.1 spike protein (PDB ID: 7TM0 (ref. ^61^)). A model of the COV2-3835 Fab molecule was generated using SAbPred ABodyBuilder^62^ and fit into the local refinement volume via ChimeraX^59^ The structure was iteratively refined and completed using a combination of Phenix (v.1.21.1)^63,64^, Coot (v.0.9.2)^65^ and ISOLDE (v.1.8)^66^. Data collection and refinement statistics are available in Supplementary Table 4.

### Cryo-EM sample and grid preparation for the BQ.1.1 spike protein in complex with COV2-3891 Fab molecules

After incubating for 2 h at room temperature, the BQ.1.1 VFLIP S-COV2-3891 Fab mixture was purified by gel filtration on Superose 6 Increase 10/300 column (GE Healthcare) equilibrated in a buffer containing 20 mM HEPES, 150 mM NaCl and 1 mM EDTA (pH 8.0). Of the purified mixture at a concentration of ~0.3 mg ml^−1^, 2.2 µl was applied to glow-discharged grids (40 s at 25 mA) (carbon grid, 300-mesh 1.2/1.3, Quantifoil). The grids were blotted for 3 s before plunging into liquid ethane using Vitrobot MK4 (Thermo Fisher) at 20 °C and 100% relative humidity. Grids were screened on a Glacios (Thermo Fisher) microscope operated at 200 keV and equipped with a Falcon 4 (Thermo Fisher) DED detector. Data were collected and imaged on a Krios (Thermo Fisher) microscope operated at 300 keV and equipped with a K3 and GIF energy filter with a 20 eV slit (Gatan) DED detector using counting mode. Datasets were collected using EPU and videos were collected at a nominal magnification of ×130,000 and pixel size of 0.647 Å pixel^−1^, with a defocus range of 0.8–1.8 µm. Grids were exposed at ~1.27 e Å^−2^ frame^−1^, resulting in a total dose of ~63 e Å^−2^ (Extended Data Fig. 7 and Supplementary Table 5).

### Cryo-EM data processing and structure building for BQ.1.1:COV2-3891 Fab complexes

To overcome the problem of preferred orientation, multiple datasets were collected with varying tilt angles (0° and 30°). These datasets were preprocessed separately and combined at a later stage. Videos were preprocessed with Relion Motioncor2 (ref. ^67^) and CTFFind4 (ref. ^68^) on the fly. Micrographs with low resolution, high astigmatism and high defocus were removed from the data set. The datasets were first picked manually and extracted in a binned box (3.0328 Å pixel^−1^). The particles were used for 2D classification, and good classes were used for training, repicking with Topaz^69^ and extraction. These particles were subjected to multiple rounds of 2D class averages, 3D initial maps and 3D classification to obtain a clean, homogeneous particle set. At this point, the datasets were combined and re-extracted at a pixel size 0.9705 Å pixel^−1^. The data were subjected to 3D autorefinement and post processing, resulting in a 3.7 Å map. The data were then subjected to CTFrefine within Relion^70,71^, and one more 3D autorefinement and post-processing step resulted in a final map at 3.6 Å resolution. Focused refinement of the RBD–Fab complexes was done on subtracted particles in Relion and cryoSPARC^56,70,71^, and the 3D autorefinement and post processing at this step resulted in a ~4.1 Å map. Sharpening was carried out using DeepEMhancer^60^. Reported resolutions are based on the gold-standard Fourier shell correlation (FSC) criteria of 0.143. Workflow schematics and detailed statistics are provided in Extended Data Fig. 7 and Supplementary Table 5. PDB ID 8FXC ref. ^72^ was used as an initial model for the RBD. ImmuneBuilder^73^ prediction was used as an initial model for the Fv of the Fab molecule. The models were first docked into the map with ChimeraX^57^. To improve the coordinates, the model was subjected to iterative refinement comprising manual building in Coot^65^ and real space refinement with Phenix^63^. The model was validated with Molprobity^74^ (Supplementary Table 5). Figures were generated using PyMOL^75^, and the EM maps and models have been deposited in the EMDB and PDB repositories (Supplementary Table 5).

### Crystallization of the XBB.1.5 RBD in complex with COV2-3906 Fab molecules

The Prescission 3C protease tag was removed from purified XBB.1.5 RBD by adding Prescission Protease (Trialtus Biosciences) to the RBD at a 1:200 mass ratio and incubating for 24 h at 4 °C. Untagged XBB.1.5 RBD was mixed with purified, recombinantly expressed COV2-3906 Fab molecules at a 1.5:1 molar ratio (RBD:Fab) and incubated for ~1 h before being loaded onto a HiLoad 16/600 Superdex 200 size exclusion column (Cytiva Life Sciences) for purification. The purified complex was concentrated to 10 mg ml^−1^ in 25 mM HEPES pH 7.3 and 100 mM NaCl, and crystallization screens were set up using a mosquito XTAL3 crystallization robot (SPT Labtech). Further optimization used the dragonfly crystal liquid handler (SPT Labtech). The XBB.1.5 RBD/COV2-3906 Fab complex was crystallized in 1.4 M ammonium sulfate, 0.1 M sodium acetate pH 5.1 and 2 mM DL-panthenol, and was cryoprotected using well solution:glycerol at a 7:3 ratio. Diffraction data were collected at the European Synchrotron Radiation Facility (ESRF) beamline ID30A-3. The data were integrated using XDS^76^ and scaled using AIMLESS^77^. The crystal structure was solved by molecular replacement with the software Phaser^78^, using Alphafold2 (refs. ^79–81^)-generated models of the XBB.1.5 RBD and COV2-3906 Fab molecule as the search models. Iterative refinement of the structure was completed using Phenix^63^ and Coot^65^. Data collection and refinement statistics are shown in Supplementary Table 6.

### Statistics and reproducibility

A non-parametric Kruskal–Wallis test with Dunn’s post hoc correction for multiple comparisons was used for comparisons between experimental conditions and the isotype-matched negative control for animal protection studies, and associated *P* values are reported without assigning thresholds for statistical significance. Data distributions were assumed to be normal but this was not formally tested. No data were excluded from analyses. Statistical methods were not used to predetermine sample sizes, but animal study sample sizes were powered on the basis of previous publications^18^. Experiments were not randomized, and investigators were not blinded to sample or participant allocation during experiments and outcome assessment.

### Reporting summary

Further information on research design is available in the Nature Portfolio Reporting Summary linked to this article.

## Supplementary information

Supplementary Information Supplementary Tables 1, 3–6 and 8.

Reporting Summary

Supplementary Table 2 SARS-CoV-2 mAb sequence features, antigen reactivity and neutralization.

Supplementary Table 7 SARS-CoV-2 mAb sequence features and antigen reactivity from additional participants.

## Source data

Source Data Fig. 2 Source data for Fig. 2b.

Source Data Fig. 3 Source data for Fig. 3b,c.

Source Data Fig. 6 Source data for Fig. 6b–d.

Source Data Extended Data Fig. 1 Source data for Extended Data Fig. 1b.

Source Data Extended Data Fig. 3 Source data.

Source Data Extended Data Fig. 4 Source data.

## Data Availability

Sequences of mAb heavy- and light-chain variable regions have been deposited in Genbank (accession numbers PP926527–PP927004, PV941706–PV941745), and sequence features and functional data from antibody screening are available in Supplementary Tables 2 and 7. The sequences of recombinant antigens are available in Supplementary Table 8. The negative-stain electron microscopy data of the Fab:spike complexes have been deposited in the Electron Microscopy Data Bank (EMDB) with the accession numbers EMD-43882 through EMD-43888. Cryo-EM data for the focused refinement of the COV2-3835-RBD interaction have been deposited in the Protein Data Bank (PDB) with the accession number 9NVG. Cryo-EM data for the BQ.1.1 spike protein-COV2-3891 Fab complex have been deposited in the EMDB with the accession code EMD-45286, and cryo-EM data for the focused refinement of the COV2-3891 Fab-RBD interaction have been deposited in the EMDB and PDB with the accession numbers EMD-45287 and 9C7S, respectively. Crystallographic data for the XBB.1.5 RBD and COV2-3906 Fab complex have been deposited in the PDB with the accession number 9C6Y. The following structures were obtained from the PDB and used for visualization or modelling: 6M0J, 6VSB, 7LRT,12E8 and 8FXC. All relevant data for each figure are available within the figures, in supplementary data, or provided as source data. No new code was generated in this study. Code used to analyse antibody sequences is available in GitHub at https://github.com/crowelab/PyIR (ref. ^82^). Further information and requests for resources and reagents should be directed to and will be fulfilled by the Lead Contact, James E. Crowe, Jr (james.crowe@vumc.org). Materials described in this paper are available for distribution for non-profit use using templated documents from the Association of University Technology Managers ‘Toolkit MTAs’, available at https://autm.net/surveys-and-tools/agreements/material-transferagreements/mta-toolkit. Source data are provided with this paper.

## Extended data

**Extended Data Table 1:** Half-maximal inhibitory concentrations (IC_50_) for authentic SARS-CoV-2 neutralization

IC50 values (ng/mL) for indicated strain				
**mAb**	WA1/2020 D614G	XBB.1.5	EG.5.1	BQ.1.1
rCOV2-3967	8.1	215.7	108.0	>
rCOV2-3872	16.5	74.2	49.4	>
rCOV2-3889	9.3	292.1	192.0	>
rCOV2-4094	30.3	183.9	158.4	>
rCOV2-3891	20.9	805.7	633.4	41.2
rCOV2-3892	476.1	629.5	652.3	1,709
rCOV2-3906	1,122	2,474	1,412	3,105
**Controls**				
rSA55	147.0	93.4	98.6	143.2
rLY-CoV1404	28.7	>	>	>
rDENV-2D22	>	>	>	>

>; indicates (IC50) value greater than 10,000 ng/mL

Calculated IC_50_ values (ng/mL) for each indicated strain for neutralization curves shown in Fig. 3b. The > symbol indicates greater than 10,000 ng/ml IC_50_ values.

**Extended Data Table 2:** Demographic data and SARS-CoV-2 exposure histories for subjects

Subject	Sex	Age at sample collection	Month of sample collection	Self-reported infection history	Self-reported vaccination history
D2102	Female	29	02/2022	12/2021 infection (likely BA.1 or BA.1.1)	Original 2 dose mRNA vaccine series (Wuhan-1), booster (Wuhan-1)
D1672	Male	33	10/2024	05/2022 infection (sequence- confirmed BA.2.12.1)	Original 2 dose mRNA vaccine series (Wuhan-1, 01/2021), booster (Wuhan-1, 10/2021), booster (Wuhan-1/BA.4 bivalent, 09/2022), booster (XBB, 10/2023)
D2015	Male	38	10/2024	01/2024 infection (sequence-confirmed JN.1)	Original 2 dose mRNA vaccine series (Wuhan-1, 2021), booster (Wuhan-1, 2021), booster (Wuhan-1/BA.4 bivalent, 2022), booster (XBB, 2023)
D2098	Male	35	10/2024	06/2024 infection (sequence-confirmed KP.3.1.1)	Original 2 dose mRNA vaccine series (Wuhan-1, 01/2021), booster (Wuhan-1, 10/2021), booster (Wuhan-1/BA.4 bivalent, 09/2022), booster (XBB, 10/2023)
D2170	Male	28	10/2024	No self-reported infection history	5 vaccine doses received

Demographic data and SARS-CoV-2 exposure histories for human subjects used for immune profiling and mAb isolation.

## Code availability

No new code was generated in this study. Code used to analyse antibody sequences is available in GitHub at https://github.com/crowelab/PyIR (ref. ^82^).
