# Supplementary material for: Epitope-focused discovery of SARS-CoV-2 antibodies that potently neutralize Omicron variants
Source: Nat Microbiol. 2026 Mar 12;11(4):1113–32. doi: 10.1038/s41564-026-02282-x (PMC13056569; doi:10.1038/s41564-026-02282-x)
Supplement: Supplementary file 1 — Supplementary Tables 1, 3–6 and 8. [file 41564_2026_2282_MOESM1_ESM.pdf]

nature microbiology

Supplementary information https://doi.org/10.1038/s41564-026-02282-x

Epitope-focused discovery of SARS-CoV-2 antibodies that potently neutralize Omicron variants

In the format provided by the authors and unedited

Supplementary Table 1. Quantification of band intensity for Coomassie-stained SDS-PAGE wth SARS-CoV-2 glycan-masked antigens

Lane	Protein loaded in lane	Amount loaded (after extinction coefficient correction)	Area (calculated using ImageJ)
1	COV2-RBD_His BA.1	2 µg	1619.548
2	COV2-RBD_His BA.1_444glyc	2 µg	1723.865
3	COV2-RBD_His BA.1_444glyc	1 µg	615.552
4	COV2-RBD Strep_BA.1	2 µg	1588.332
5	COV2-RBD_Strep BA.1_444glyc	2 µg	1090.136
6	COV2-RBD_Strep BA.1_444glyc	1 µg	536.113
7	COV2-RBD_His XBB	2 µg	2189.532
8	Ladder	Spectra multicolor protein ladder (Cat.no:3131249)	
9	BSA	2 µg	15382.338
10	BSA	1 µg	14579.388
11	BSA	0.5 µg	6702.347
12	BSA	0.25 µg	3795.255

!

Supplementary Table 3. Summary of NS-EM data collection and statistics for S protein complexes with Fabs

	Spike antigen (VFLIP)	**XBB**	**XBB**	**BQ 1.1**	**XBB**	**XBB**	**XBB**	**XBB**
**Microscope setting**	Fab	**COV2-3872**	**COV2-3889**	**COV2-3891**	**COV2-3892**	**COV2-3906**	**COV2-3967**	**COV2-4094**
	EMDB Accession #	**EMD-43882**	**EMD-43883**	**EMD-43884**	**EMD-43885**	**EMD-43886**	**EMD-43887**	**EMD-43888**
	Microscope	TF-20	TF-20	TF-20	TF-20	TF-20	TF-20	TF-20
	Voltage (kV)	200	200	200	200	200	200	200
	Detector	US-4000 CCD	US-4000 CCD	US-4000 CCD	US-4000 CCD	US-4000 CCD	US-4000 CCD	US-4000 CCD
	Magnification	x50000	x50000	x50000	x50000	x50000	x50000	x50000
	Pixel size	2.18	2.18	2.18	2.18	2.18	2.18	2.18
	Exposure (e-/Å2)	30	30	30	30	30	30	30
	Defocus range (μm)	1.5-1.8	1.5-1.8	1.5-1.8	1.5-1.8	1.5-1.8	1.5-1.8	1.5-1.8
**Data**	Micrographs (#)	146	212	250	300	296	357	222
	Particles (#)	22,418	34,112	36,000	16,171	17,006	59,482	21,505
	Particles (#) after 2D	22,418	21,963	30,118	13,238	13,559	11,541	18,343
	Final particles (#)	13,449	12,255	10,767	11,178	6,742	6,675	14,240
	Symmetry	C3	C1	C1	C1	C3	C1	C3
**Model docking**	Spike	PDB: 7LRT						
	Fab	PDB: 12E8						

Supplementary Table 4. Data collection and refinement statistics for the cryoEM structure of the BA.1 spike + COV2-3835 Fab complex.

**Sample**	SARS-CoV-2 BA.1 S6P (HexaPro) + COV2-3835 Fab	
**Cryo-EM data collection**		
Microscope	FEI Titan Krios	
Voltage (kV)	300	
Detector	Gatan K3	
Energy filter	Gatan Biocontinuum	
Slit width (eV)	20	
Magnification (nominal)	105,000	
Pixel size (Å/pix)	0.84	
Exposure rate (e^-/pix/sec)	14	
Exposure (e^-/Å^2)	60	
Defocus range (mm)	1.0-2.5	
Tilt angle (°)	0	
Micrographs collected	3,291	
Micrographs used	3,096	
Particles extracted	1,127,216	
Automation software	SerialEM	
**3D reconstruction statistics**		
Refinement	Local (RBD + Fv)	Global
Particles	152,740	152,740
Symmetry	C1	C1
Map sharpening B-factor	-79.2	-64.7
Resolution (Å) at FSC (half-maps)		
Unmasked: 0.5	9.3	6.9
Masked: 0.5	3.3	3.1
Unmasked: 0.143	4.3	3.6
Masked: 0.143	3.0	2.8
EMDB ID	49835	49799
**Model refinement and validation statistics**		
Composition		
Amino Acids	413	
Ligands (type)	2 (NAG)	
Bonds (RMSD)		
Length (Å) (# > 4σ)	0.003 (0)	
Angles (°) (# > 4σ)	0.63 (0)	
Ramachandran plot		
Outliers (%)	0.0	
Allowed (%)	0.0	
Favored (%)	100.0	
Rotamer outliers (%)	0.0	
C-b outliers (%)	0.0	
CaBLAM outliers (%)	0.77	
ADP (B-factors)		
Amino Acids (mean)	43.4	
Ligands (mean)	86.8	
CC (mask)	0.89	
MolProbity score	0.78	
Clash score	0.93	
EMRinger score	4.69	
PDB ID	9NVG	

Supplementary Table 5. Data collection and refinement statistics for the cryoEM structure of the BQ.1.1 spike + COV2-3891 Fab complex.

		BQ.1.1 COV2-3891	BQ.1.1 RBD COV2-3891
Data Deposition	EMDB	EMD-45286	EMD-45287
	PDB	--	9C7S
Microscope setting	Microscope	Krios	Krios
	Voltage (kV)	300	300
	Detector	K3	K3
	Mag	130000	130000
	Pixel size	0.647	0.647
	Exposure (e-/Å^2)	58	58
	Defocus range (μm)	0.8-1.8	0.8-1.8
Data	# Micrographs	14850	14850
	# particles	400000	400000
	# particle after 2D	375000	375000
	Final particles #	167055	167055
	Symmetry	C1	C1
	Map B factor	89.3	162
	Map resolution (Å) FSC=0.143	3.6	4.05
Model refinement and validation	Initial model used		Ab-initio
	Model resolution (Å) FSC=0.5		4.2
	Protein residues		491
	Ligand		2
	Map CC		0.67
	RMSD		
	Bond lengths (Å)		0.003
	Bond angles		0.806
	Ramachandran		
	Outliers (%)		0
	Allowed (%)		4.95
	Favored (%)		95.05
	Poor rotamers (%)		0.24
	MolProbity score		2.17
	Clash score		21.77
	CaBLAM score		1.25
	B factors (Å^2)		
	Protein		71/163/102
	Ligand		98/145/121

Supplementary Table 6. Data collection and refinement statistics for the crystal structure of the XBB.1.5 RBD + COV2-3906 Fab complex.

	RBD:COV2-3906 Fab
**PDB ID**	9C6Y
**Data collection**	
Space group	$P2_12_12_1$
a, b, c (Å)	66.9, 171, 232
α, β, γ (°)	90, 90, 90
Resolution (Å)	48.5 - 2.68 (2.77-2.68)
R_{merge}	0.217 (3.117)
CC1/2	0.996 (0.492)
I/σ	10.7 (0.9)
Completeness (%)	99.9 (100)
Redundancy	13.8 (14.5)
**Refinement**	
No. unique reflections	75613 (7276)
R_{work}/R_{free}	0.207/0.242
No. atoms	
Protein	9810
Ligand/ion	192
Water	94
B-factors	
Protein	67.32
Ligand/ion	92.90
Water	59.56
R.m.s deviations	
Bond length (Å)	0.010
Bond angle (°)	1.067
Ramanchandran	
Favored (%)	94.54
Allowed (%)	4.84
Outliers (%)	0.62

*Values in parentheses are for the highest resolution shell

Supplementary Table 8. Sequences of recombinant antigens used for binding assays, structural studies, and antigen-specific staining

COV2-RBD_His WT
MGILPSPGMPALLSLVSLLSVLLMGCVAETGTRFPNITNLCPFGEVFNATRFASVYAWNRKRISNCVADFS
VLYNSASFSTFKCYGVSPTKLNDLCWTNIYADSFVIRGDEVRQIAPGQTGKIADYNYKLPDDFTGCVIAWN
SNNLDSKVGGNYNYLYRLFRKSNLKPFERDISTEIYQAGSTPCNGVEGFNCYFPLQSYGFQPTNGVGYQ
PYRVVVLSFELLHAPATVCGPKKSTGLNDIFEAQKIEWHEHHHHHHHH*

COV2-RBD_His BA.1
MGILPSPGMPALLSLVSLLSVLLMGCVAETGTRFPNITNLCPFDEVFNATRFASVYAWNRKRISNCVADFS
VLYNLAPFFTFKCYGVSPTKLNDLCWTNIYADSFVIRGDEVRQIAPGQTGNIADYNYKLPDDFTGCVIAWN
SNKLDSKVSGNYNYLYRLFRKSNLKPFERDISTEIYQAGNKPCNGVAGFNCYFPLRSYSFRPTYGVGHQP
YRVVVLSFELLHAPATVCGPKKSTGLNDIFEAQKIEWHEHHHHHHHH*

COV2-RBD_His BA.1_444glyc
MGILPSPGMPALLSLVSLLSVLLMGCVAETGTRFPNITNLCPFDEVFNATRFASVYAWNRKRISNCVADFS
VLYNLAPFFTFKCYGVSPTKLNDLCWTNIYADSFVIRGDEVRQIAPGQTGNIADYNYKLPDDFTGCVIAWN
SNKLDSNVTGNYNYLYRLFRKSNLKPFERDISTEIYQAGNKPCNGVAGFNCYFPLRSYSFRPTYGVGHQP
YRVVVLSFELLHAPATVCGPKKSTGLNDIFEAQKIEWHEHHHHHHHHDYKDDDDK*

COV2-RBD_His BA.2
MGILPSPGMPALLSLVSLLSVLLMGCVAETGTRFPNITNLCPFDEVFNATRFASVYAWNRKRISNCVADFS
VLYNFAPFFAFKCYGVSPTKLNDLCWTNIYADSFVIRGNEVSQIAPGQTGNIADYNYKLPDDFTGCVIAWN
SNKLDSKVGGNYNYLYRLFRKSNLKPFERDISTEIYQAGNKPCNGVAGFNCYFPLRSYGFRPTYGVGHQ
PYRVVVLSFELLHAPATVCGPKKSTGLNDIFEAQKIEWHEHHHHHHHH*

COV2-RBD_His BQ.1.1
MGILPSPGMPALLSLVSLLSVLLMGCVAETGTRFPNITNLCPFDEVFNATTFASVYAWNRKRISNCVADFSV
LYNFAPFFAFKCYGVSPTKLNDLCWTNIYADSFVIRGNEVSQIAPGQTGNIADYNYKLPDDFTGCVIAWNS
NKLDSTVGGNYNYRYRLFRKSKLKPFERDISTEIYQAGNKPCNGVAGVNCYFPLQSYGFRPTYGVGHQP
YRVVVLSFELLHAPATVCGPKKSTGLNDIFEAQKIEWHEHHHHHHHHEQKLISEEDL*

COV2-RBD_His XBB
MGILPSPGMPALLSLVSLLSVLLMGCVAETGTRFPNITNLCPFHEVFNATTFASVYAWNRKRISNCVADFSV
IYNFAPFFAFKCYGVSPTKLNDLCWTNIYADSFVIRGNEVSQIAPGQTGNIADYNYKLPDDFTGCVIAWNS
NKLDSKPSGNYNYLYRLFRKSKLKPFERDISTEIYQAGNKPCNGVAGSNCYSPLQSYGFRPTYGVGHQP
YRVVVLSFELLHAPATVCGPKKSTGLNDIFEAQKIEWHEHHHHHHHHEQKLISEEDL*

COV2-RBD_Strep BA.1
MYRMQLLSCIALSLALVTNSRVQPTESIVRFPNITNLCPFDEVFNATRFASVYAWNRKRISNCVADFSVLYN
LAPFFTFKCYGVSPTKLNDLCWTNIYADSFVIRGDEVRQIAPGQTGNIADYNYKLPDDFTGCVIAWNSNKL
DSKVSGNYNYLYRLFRKSNLKPFERDISTEIYQAGNKPCNGVAGFNCYFPLRSYSFRPTYGVGHQPYRVV
VLSFELLHAPATVCGPKLEVLFQGPGGLNDIFEAQKIEWHEGENLYFQGSAWSHPQFEKGGGSGGGGS
GGSAWSHPQFEKHHHHHHHH*

COV2-RBD_Strep BA.1_444glyc
MYRMQLLSCIALSLALVTNSRVQPTESIVRFPNITNLCPFDEVFNATRFASVYAWNRKRISNCVADFSVLYN
LAPFFTFKCYGVSPTKLNDLCWTNIYADSFVIRGDEVRQIAPGQTGNIADYNYKLPDDFTGCVIAWNSNKL
DSNVTGNYNYLYRLFRKSNLKPFERDISTEIYQAGNKPCNGVAGFNCYFPLRSYSFRPTYGVGHQPYRVV

VLSFELLHAPATVCGPKLEVLFQGPGGLNDIFEAQKIEWHEGENLYFQGSAWSHPQFEKGGGSGGGGS
GGSAWSHPQFEKHHHHHHHH*

COV2-RBD_Strep XBB.1.5

MYRMQLLSCIALSLALVTNSRVQPTESIVRFPNITNLCPFHEVFNATTFASVYAWNRKRISNCVADFSVIYN
FAPFFAFKCYGVSPTKLNDLCWTNIYADSFVIRGNEVSQIAPGQTGNIADYNYKLPDDFTGCVIAWNSNKL
DSKPSGNYNYLYRLFRKSKLKPFERDISTEIYQAGNKPCNGVAGPNCYSPLQSYGFRPTYGVGHQPYRV
VVLSFELLHAPATVCGPKLEVLFQGPGGLNDIFEAQKIEWHEGENLYFQGSAWSHPQFEKGGGSGGGGS
GGSAWSHPQFEKHHHHHHHH*

BA.2 S_VFLIP

MFVFLVLLPLVSSQCVNLITRTQSYTNSFTRGVYYPDKVFRSSVLHSTQDLFLPFFSNVTWFHAIHVSGTN
GTKRFDNPVLPFNDGVYFASTEKSNIIRGWIFGTTLDSKTQSLLIVNNATNVVIKVCEFQFCNDPFLDVYYH
KNNKSWMESEFRVYSSANNCTFEYVSQPFLMDLEGKQGNFKNLREFVFKNIDGYFKIYSKHTPINLGRDL
PQGFSALEPLVDLPIGINITRFQTLLALHRSYLTPGDSSSGWTAGAAAYYVGYLQPRTFLLKYNENGTITDA
VDCALDPLSETKCTLKSFTVEKGIYQTSNFRVQPTESIVRFPNITNLCPFDEVFNATRFASVYAWNRKRISN
CVADYSVLYNFAPFFAFKCYGVSPTKLNDLCFTNVYADSFVIRGNEVSQIAPGQTGNIADYNYKLPDDFTG
CVIAWNSNKLDSKVGGNYNYLYRLFRKSNLKPFERDISTEIYQAGNKPCNGVAGFNCYFPLRSYGFRPTY
GVGHQPYRVVVLSFELLHAPATVCGPKKSTNLVKNKCVNFNFNGLKGTGVLTESNKKFLPFQQFGRDIAD
TTDAVRDPQTLEILDITPCSFGGVSVITPGTNTSNQVAVLYQGVNCTEVPVAIHADQLTPTWRVYSTGSNV
FQTRAGCLIGAEYVNNSYECDIPIGAGICASYQGGGSGGGSSIIAYTMSLGAENSVACSNNSIAIPTNFTISV
TTEILPVSMTKTSVDCTMYICGDSTECSNLLLQYGSFCTQLKRALTGIAVEQDKNTQEVFAQVKQIYKTPPI
KYFGGFNFSQILPDPSKPSKRSPIEDLLFNKVTLADAGFIKQYGDCLGDIAARDLICAQKFNGLTVLPPLLTD
EMIAQYTSALLAGTICSGWTFGAGPALQIPFPMQMAYRFNGIGVTQNVLYENQKLIANQFNSAIGKIQDSLS
STPSALGKLQDVVNHNAQALNTLVKQLSSKFGAISSVLNDFLSRLDKPEAEVQIDRLITGRLQSLQTYVTQ
QLIRAAEIRASANLAATKMSECVLGQSKRVDFCGKGYHLMSFPQSAPHGVVFLHVTYVPAQEKNFTTAPAI
CHDGKAHFPREGVFVSNGTHWFVTQRNFYEPQIITTDNTFVSGNCDVVIGIVNNTVYDPLQPELDSFKEE
LDKYFKNHTSPDVDLGDISGINASVVNIQKEIDRLNEVAKNLNESLIDLQELGKYEQGSGYIPEAPRDGQAY
VRKDGEWVLLSTFLGRSLEVLFQGPGHHHHHHHHSAWSHPQFEKGGGSGGGGSGGSAWSHPQFEK*

BQ.1.1 S_VFLIP

MFVFLVLLPLVSSQCVNLITRTQSYTNSFTRGVYYPDKVFRSSVLHSTQDLFLPFFSNVTWFHAISGTNGT
KRFDNPVLPFNDGVYFASTEKSNIIRGWIFGTTLDSKTQSLLIVNNATNVVIKVCEFQFCNDPFLDVYYHKN
NKSWMESEFRVYSSANNCTFEYVSQPFLMDLEGKQGNFKNLREFVFKNIDGYFKIYSKHTPINLGRDLPQ
GFSALEPLVDLPIGINITRFQTLLALHRSYLTPGDSSSGWTAGAAAYYVGYLQPRTFLLKYNENGTITDAVD
CALDPLSETKCTLKSFTVEKGIYQTSNFRVQPTESIVRFPNITNLCPFDEVFNATTFASVYAWNRKRISNCV
ADYSVLYNFAPFFAFKCYGVSPTKLNDLCFTNVYADSFVIRGNEVSQIAPGQTGNIADYNYKLPDDFTGCV
IAWNSNKLDSTVGGNYNYRYRLFRKSKLKPFERDISTEIYQAGNKPCNGVAGVNCYFPLQSYGFRPTYGV
GHQPYRVVVLSFELLHAPATVCGPKKSTNLVKNKCVNFNFNGLTGTGVLTESNKKFLPFQQFGRDIADTT
DAVRDPQTLEILDITPCSFGGVSVITPGTNTSNQVAVLYQGVNCTEVPVAIHADQLTPTWRVYSTGSNVFQ
TRAGCLIGAEYVNNSYECDIPIGAGICASYQGGGSGGGSSIIAYTMSLGAENSVACSNNSIAIPTNFTISVTT
EILPVSMTKTSVDCTMYICGDSTECSNLLLQYGSFCTQLKRALTGIAVEQDKNTQEVFAQVKQIYKTPPIKY
FGGFNFSQILPDPSKPSKRSPIEDLLFNKVTLADAGFIKQYGDCLGDIAARDLICAQKFNGLTVLPPLLTDE
MIAQYTSALLAGTICSGWTFGAGPALQIPFPMQMAYRFNGIGVTQNVLYENQKLIANQFNSAIGKIQDSLSS
TPSALGKLQDVVNHNAQALNTLVKQLSSKFGAISSVLNDILSRLDKPEAEVQIDRLITGRLQSLQTYVTQQL
IRAAEIRASANLAATKMSECVLGQSKRVDFCGKGYHLMSFPQSAPHGVVFLHVTYVPAQEKNFTTAPAIC
HDGKAHFPREGVFVSNGTHWFVTQRNFYEPQIITTDNTFVSGNCDVVIGIVNNTVYDPLQPELDSFKEEL
DKYFKNHTSPDVDLGDISGINASVVNIQKEIDRLNEVAKNLNESLIDLQELGKYEQGSGYIPEAPRDGQAYV
RKDGEWVLLSTFLGRSLEVLFQGPGHHHHHHHHSAWSHPQFEKGGGSGGGGSGGSAWSHPQFEK*

XBB S_VFLIP
MFVFLVLLPLVSSQCVNLITRTQSYTNSFTRGVYYPDKVFRSSVLHSTQDLFLPFFSNVTWFHAIHVSGTN
GTKRFDNPALPFNDGVYFASTEKSNIIRGWIFGTTLDSKTQSLLIVNNATNVVIKVCEFQFCNDPFLDVYQK
NNKSWMESEFRVYSSANNCTFEYVSQPFLMDLEGKEGNFKNLREFVFKNIDGYFKIYSKHTPINLERDLP
QGFSALEPLVDLPIGINITRFQTLLALHRSYLTPGDSSSGWTAGAAAYYVGYLQPRTFLLKYNENGTITDAV
DCALDPLSETKCTLKSFTVEKGIYQTSNFRVQPTESIVRFPNITNLCPFHEVFNATTFASVYAWNRKRISNC
VADYSVIYNFAPFFAFKCYGVSPTKLNDLCFTNVYADSFVIRGNEVSQIAPGQTGNIADYNYKLPDDFTGC
VIAWNSNKLDSKPSGNYNYLYRLFRKSKLKPFERDISTEIYQAGNKPCNGVAGSNCYSPLQSYGFRPTYG
VGHQPYRVVVLSFELLHAPATVCGPKKSTNLVKNKCVNFNFNGLTGTGVLTESNKKFLPFQQFGRDIADT
TDAVRDPQTLEILDITPCSFGGVSVITPGTNTSNQVAVLYQGVNCTEVPVAIHADQLTPTWRVYSTGSNVF
QTRAGCLIGAEYVNNSYECDIPIGAGICASYQGGGSGGGSSIIAYTMSLGAENSVACSNNSIAIPTNFTISVT
TEILPVSMTKTSVDCTMYICGDSTECSNLLLQYGSFCTQLKRALTGIAVEQDKNTQEVFAQVKQIYKTPPIK
YFGGFNFSQILPDPSKPSKRSPIEDLLFNKVTLADAGFIKQYGDCLGDIAARDLICAQKFNGLTVLPPLLTDE
MIAQYTSALLAGTICSGWTFGAGPALQIPFPMQMAYRFNGIGVTQNVLYENQKLIANQFNSAIGKIQDSLSS
TPSALGKLQDVVNHNAQALNTLVKQLSSKFGAISSVLNDILSRLDKPEAEVQIDRLITGRLQSLQTYVTQQL
IRAAEIRASANLAATKMSECVLGQSKRVDFCGKGYHLMSFPQSAPHGVVFLHVTYVPAQEKNFTTAPAIC
HDGKAHFPREGVFVSNGTHWFVTQRNFYEPQIITTDNTFVSGNCDVVIGIVNNTVYDPLQPELDSFKEEL
DKYFKNHTSPDVDLGDISGINASVVNIQKEIDRLNEVAKNLNESLIDLQELGKYEQGSGYIPEAPRDGQAYV
RKDGEWVLLSTFLGRSLEVLFQGPGHHHHHHHHSAWSHPQFEKGGGSGGGGSGGSAWSHPQFEK*

SARS-CoV (S-2P)
MFIFLLFLTLTSGSDLDRCTTFDDVQAPNYTQHTSSMRGVYYPDEIFRSDTLYLTQDLFLPFYSNVTGFHTI
NHTFGNPVIPFKDGIYFAATEKSNVVRGWVFGSTMNNKSQSVIIINNSTNVVIRACNFELCDNPFFAVSKP
MGTQTHTMIFDNAFNCTFEYISDAFSLDVSEKSGNFKHLREFVFKNKDGFLYVYKGYQPIDVVRDLPSGF
NTLKPIFKLPLGINITNFRAILTAFSPAQDIWGTSAAAYFVGYLKPTTFMLKYDENGTITDAVDCSQNPLAELK
CSVKSFEIDKGIYQTSNFRVVPSGDVVRFPNITNLCPFGEVFNATKFPSVYAWERKKISNCVADYSVLYNS
TFFSTFKCYGVSATKLNDLCFSNVYADSFVVKGDDVRQIAPGQTGVIADYNYKLPDDFMGCVLAWNTRNI
DATSTGNYNYKYRYLRHGKLRPFERDISNVPFSPDGKPCTPPALNCYWPLNDYGFYTTTGIGYQPYRVVV
LSFELLNAPATVCGPKLSTDLIKNQCVNFNFNGLTGTGVLTPSSKRFQPFQQFGRDVSDFTDSVRDPKTS
EILDISPCAFGGVSVITPGTNASSEVAVLYQDVNCTDVSTAIHADQLTPAWRIYSTGNNVFQTQAGCLIGAE
HVDTSYECDIPIGAGICASYHTVSLLRSTSQKSIVAYTMSLGADSSIAYSNNTIAIPTNFSISITTEVMPVSMA
KTSVDCNMYICGDSTECANLLLQYGSFCTQLNRALSGIAAEQDRNTREVFAQVKQMYKTPTLKYFGGFN
FSQILPDPLKPTKRSFIEDLLFNKVTLADAGFMKQYGECLGDINARDLICAQKFNGLTVLPPLLTDDMIAAYT
AALVSGTATAGWTFGAGAALQIPFAMQMAYRFNGIGVTQNVLYENQKQIANQFNKAISQIQESLTTTSTAL
GKLQDVVNQNAQALNTLVKQLSSNFGAISSVLNDILSRLDPPEAEVQIDRLITGRLQSLQTYVTQQLIRAAE
IRASANLAATKMSECVLGQSKRVDFCGKGYHLMSFPQAAPHGVVFLHVTYVPSQERNFTTAPAICHEGKA
YFPREGVFVFNGTSWFITQRNFFSPQIITTDNTFVSGNCDVVIGIINNTVYDPLQPELDSFKEELDKYFKNH
TSPDVDLGDISGINASVVNIQKEIDRLNEVAKNLNESLIDLQELGKYEQGSGYIPEAPRDGQAYVRKDGEW
VLLSTFLGRSLEVLFQGPGHHHHHHHHSAWSHPQFEK*
