# Supplementary material for: Epitope-focused discovery of SARS-CoV-2 antibodies that potently neutralize Omicron variants
Source: Nat Microbiol. 2026 Mar 12;11(4):1113–32. doi: 10.1038/s41564-026-02282-x (PMC13056569; doi:10.1038/s41564-026-02282-x)
Supplement: Supplementary file 2 — Reporting Summary [file 41564_2026_2282_MOESM2_ESM.pdf]

nature portfolio

Corresponding author(s): James E. Crowe Jr., MD

Last updated by author(s): Jan 20, 2026

Reporting Summary

Nature Portfolio wishes to improve the reproducibility of the work that we publish. This form provides structure for consistency and transparency in reporting. For further information on Nature Portfolio policies, see our Editorial Policies and the Editorial Policy Checklist.

Statistics

For all statistical analyses, confirm that the following items are present in the figure legend, table legend, main text, or Methods section.

n/a	Confirmed	
☐	☒	The exact sample size (*n*) for each experimental group/condition, given as a discrete number and unit of measurement
☐	☒	A statement on whether measurements were taken from distinct samples or whether the same sample was measured repeatedly
☐	☒	The statistical test(s) used AND whether they are one- or two-sided<br>*Only common tests should be described solely by name; describe more complex techniques in the Methods section.*
☒	☐	A description of all covariates tested
☐	☒	A description of any assumptions or corrections, such as tests of normality and adjustment for multiple comparisons
☐	☒	A full description of the statistical parameters including central tendency (e.g. means) or other basic estimates (e.g. regression coefficient) AND variation (e.g. standard deviation) or associated estimates of uncertainty (e.g. confidence intervals)
☐	☒	For null hypothesis testing, the test statistic (e.g. *F*, *t*, *r*) with confidence intervals, effect sizes, degrees of freedom and *P* value noted<br>*Give P values as exact values whenever suitable.*
☒	☐	For Bayesian analysis, information on the choice of priors and Markov chain Monte Carlo settings
☒	☐	For hierarchical and complex designs, identification of the appropriate level for tests and full reporting of outcomes
☒	☐	Estimates of effect sizes (e.g. Cohen's *d*, Pearson's *r*), indicating how they were calculated

Our web collection on statistics for biologists contains articles on many of the points above.

Software and code

Policy information about availability of computer code

Data collection	xCELLigence Analyzer (Agilent) , spectrophotometer (Biotek), Titan Krios G3i (SLAC, USA), SH800 cell sorter (Sony Biotechnology), Beacon® optofluidic system (Berkeley Lights), European Synchrotron Radiation Facility (ESRF) beamline ID30A-3
Data analysis	Prism v.9.0 (GraphPad). ImmunoSpot microanalyzer (Cellular Technologies). Infectious viral loads were compared by a one-way ANOVA with Dunnett's multiple comparisons test using Prism. UCSF Chimera (RRID: SCR_004097), Coot version 0.9.8.1 EL, EPU software (Thermo Fisher Scientific) Negative stain electron microscopy image acquisition and processing was performed with cryoSPARC software package version 2.14.2 (PMID: 28165473). DeepEMhancer-v20210511 (PMID: 34267316). RTCA xCelligence data was analyzed using RTCA software pro Version 2.3.4. ChimeraX was used for model docking (PMID: 32881101). FlowJo version 10 (Tree Star).

For manuscripts utilizing custom algorithms or software that are central to the research but not yet described in published literature, software must be made available to editors and reviewers. We strongly encourage code deposition in a community repository (e.g. GitHub). See the Nature Portfolio guidelines for submitting code & software for further information.

Data

Policy information about availability of data

All manuscripts must include a data availability statement. This statement should provide the following information, where applicable:

- Accession codes, unique identifiers, or web links for publicly available datasets
- A description of any restrictions on data availability
- For clinical datasets or third party data, please ensure that the statement adheres to our policy

Sequences of mAb heavy- and light-chain variable regions have been deposited in Genbank (accession numbers PP926527-PP927004, PV941706-PV941745) and sequence features and functional data from antibody screening are available in Supplementary Table 2 and Supplementary Table 7. The sequences of recombinant antigens are available in Supplementary Table 8. The negative-stain electron microscopy data of the Fab:spike complexes have been deposited in the Electron Microscopy Data Bank (EMDB) with the accession numbers EMD-43882 through EMD-43888. Cryo-EM data for the focused refinement of the COV2-3835-RBD interaction have been deposited in the Protein Data Bank (PDB) with the accession number 9NVG. Cryo-EM data for the BQ.1.1 spike protein-COV2-3891 Fab complex have been deposited in the EMDB with the accession code EMD-45286 and cryo-EM data for the focused refinement of the COV2-3891 Fab-RBD interaction have been deposited in the EMDB and PDB with the accession numbers EMD-45287 and 9C7S, respectively. Crystallographic data for the XBB.1.5 RBD and COV2-3906 Fab complex have been deposited in the PDB with the accession number 9C6Y. The following structures were obtained from the PDB and used for visualization: 6M0J, 6VSB, 7LRT, and 12E8. All relevant data for each figure are available within the figures, in Supplementary Data, or provided as source data. No new code was generated in this study. Code used to analyze antibody sequences is available at https://github.com/crowelab/PyIR. Further information and requests for resources and reagents should be directed to and will be fulfilled by the Lead Contact, James E. Crowe, Jr. (james.crowe@vumc.org). Materials described in this paper are available for distribution for nonprofit use using templated documents from the Association of University Technology Managers ''Toolkit MTAs'', available at: https://autm.net/surveys-and-tools/agreements/material-transferagreements/mta-toolkit.

Research involving human participants, their data, or biological material

Policy information about studies with human participants or human data. See also policy information about sex, gender (identity/presentation), and sexual orientation and race, ethnicity and racism.

Reporting on sex and gender	Two females and three males
Reporting on race, ethnicity, or other socially relevant groupings	Female 1. Hispanic white, 28 y/o, female 2. White American, 28 y/o. Male 1. Asian 38 y/o, Male 2. White American 33y/o Male 3. White American 35 y/o.
Population characteristics	PBMC sample was used, with reported race, ethnicity of other social relevant grouping.
Recruitment	Donors were recruited based on their infection and vaccination status.
Ethics oversight	Vanderbilt University Medical Center Institutional Review Board

Note that full information on the approval of the study protocol must also be provided in the manuscript.

Field-specific reporting

Please select the one below that is the best fit for your research. If you are not sure, read the appropriate sections before making your selection.

☒ Life sciences ☐ Behavioural & social sciences ☐ Ecological, evolutionary & environmental sciences

For a reference copy of the document with all sections, see nature.com/documents/nr-reporting-summary-flat.pdf

Life sciences study design

All studies must disclose on these points even when the disclosure is negative.

Sample size	Sample-size calculations were not performed to power the study. Sample sizes for mouse studies were determined based on our previous results for similar in vivo experiments that showed that the use of 5 mice per group represents a minimally sufficient sample size to produce a study power of >80% (adequacy standard used in most research). See PMID: 33031744 for reference. Furthermore, the mice study sample sizes were sufficient given the large differences in viral load between treated and isotype control groups. Details about groups and sample sizes for mice virus challenge studies are provided in the results section and figure legends. No other experiments requires calculation of the power .
Data exclusions	No data were excluded from the analysis
Replication	Studies that were repeated are noted in figure captions and include all studies that demonstrated the key results reported in the manuscript. No studies have been reported that failed upon repetition. Antibodies of known activity were included across all experiments to verify reproducibility (e.g. presence of binding, blocking, or neutralizing activities), and included comparisons of newly identified SA-55 and isotype matched antibody controls. These controls were included in each replicate experiment that measured binding, blocking, neutralizing, and in vivo protective activity of characterized mAbs. Consistency of mAb activity across in vitro and in vivo experiments within this study indicate a high level of reproducibility.

Randomization	Twelve-month-old hACE2-K18 mice of both sexes were randomly allocated to the groups. For experiments other than animal studies, randomization is not relevant as this is an observational study.
Blinding	We used conventional blinding approach by including Isotype control mAbs, along with virus only and cells only controls in our pesudotyped and authentic virus neutralization assays.

Reporting for specific materials, systems and methods

We require information from authors about some types of materials, experimental systems and methods used in many studies. Here, indicate whether each material, system or method listed is relevant to your study. If you are not sure if a list item applies to your research, read the appropriate section before selecting a response.

Materials & experimental systems

n/a	Involved in the study
☐	☒ Antibodies
☐	☒ Eukaryotic cell lines
☒	☐ Palaeontology and archaeology
☐	☒ Animals and other organisms
☒	☐ Clinical data
☒	☐ Dual use research of concern
☒	☐ Plants

Methods

n/a	Involved in the study
☒	☐ ChIP-seq
☐	☒ Flow cytometry
☒	☐ MRI-based neuroimaging

Antibodies

Antibodies used	Goat Anti-Human IgG Fc, Multi-Species SP ads :2014-05 Horseradish Peroxidase (HRP) Southern biotech. Positive (rSA55 PMID: 36535326, rLY-CoV1404 (PMID: 35568025) ; isotype control mAb rDENV-2D22 (PMID: 26138979) references are provided. Antibodies (COV2-3967 , COV2-3872, COV2-3889 , COV2-4094 , COV2-3891, COV2-3892 ,COV2-3906) identified in this study are validated twice for binding and neutralization. Antibody sequences will be made available once the paper is accepted.
Validation	For antibodies identified in this study, data is provided in the manuscript.

Eukaryotic cell lines

Policy information about cell lines and Sex and Gender in Research

Cell line source(s)	Expi293F cells (Thermo Fisher Scientific; catalog number A1452) were maintained at 37°C in 8% CO2 in Expi293F Expression Medium (Thermo Fisher Scientific; catalog number A1435102). An NIH3T3 engineered fibroblast line (mouse, male origin) constitutively expressing cell-surface human CD154 (CD40 ligand), secreted human B-cell activating factor (BAFF) and human IL-21 was kindly provided by Dr. Deepta Bhattacharya (Washington University in St. Louis, MO). HEK-293T/17 cells, a subclone cell line exhibiting epithelial morphology that was isolated from human embryo kidney tissue, expressing the simian virus 40 (SV40) large T antigen, and exhibiting high transfectability was obtained from The American Type Culture Collection (ATCC; cat. CRL-11268). HEK-293T cells stably transduced to express human ACE2 (293T-hACE2 cells) were obtained from BEI Resources (cat. NR-52511). Anti-human CD45-PE (BioLegend, cat. 368509)
Authentication	None of these cell lines were authenticated
Mycoplasma contamination	All cell lines were tested negative for mycoplasma
Commonly misidentified lines (See ICLAC register)	No commonly misidentified lines were used in this study.

Animals and other research organisms

Policy information about studies involving animals; ARRIVE guidelines recommended for reporting animal research, and Sex and Gender in Research

Laboratory animals	Twelve-month-old hACE2-K18 mice of both sexes were randomly allocated to the groups.
Wild animals	Study does not involve wild animals
Reporting on sex	Twelve-month-old hACE2-K18 mice of both sexes were randomly allocated to the groups.
Field-collected samples	Study does not involve field-collected samples
Ethics oversight	Institutional Animal Care and Use Committee at UNC-Chapel Hill (protocol number 23-155)

Note that full information on the approval of the study protocol must also be provided in the manuscript.

Flow Cytometry

Plots

Confirm that:

☒ The axis labels state the marker and fluorochrome used (e.g. CD4-FITC).

☒ The axis scales are clearly visible. Include numbers along axes only for bottom left plot of group (a 'group' is an analysis of identical markers).

☒ All plots are contour plots with outliers or pseudocolor plots.

☒ A numerical value for number of cells or percentage (with statistics) is provided.

Methodology

Sample preparation	Cryopreserved PBMCs from each donor were thawed in a 37°C water bath and immediately mixed with cold RoboSepTM buffer (StemCELL Technologies, cat. 20104). After a brief centrifugation (250 xg, 5 minutes) at room temperature, the cell pellet was resuspended in cold RoboSepTM buffer. B cells were enriched using a negative-selection magnetic bead-based enrichment kit (EasySep Human B Cell Isolation Kit, StemCELL Technologies, cat. 17954) according to the manufacturer's protocol. After washing with cold RoboSepTM buffer (250 xg, 5 minutes) at room temperature, the isolated B cells were incubated with a cocktail of phenotyping antibodies.
Instrument	SH800 cell sorter (Sony Biotechnology)
Software	SH800 software (Sony Biotechnology) and FlowJo version 10 (Tree Star)
Cell population abundance	While XBB-RBD+, 444glyc-RBD+ cells were ~1.25% of total class-switched memory B cells, XBB-RBD+, 444glyc-RBD- cells were present at an even lower frequency of ~0.35% . Of the class-switched memory B cells, ~0.79% were XBB-RBD-, 444glyc-RBD+, likely representing B cells that bound the BA.1 RBD containing the 444 glycan but did not cross-react with XBB. In comparison, BQ.1.1-RBD+, 444glyc-RBD- cells were present at a frequency of ~0.22%
Gating strategy	We first enriched for B cells from peripheral blood mononuclear cells (PBMCs) using negative-selection and then stained cells with antibodies specific for CD19, IgD, and IgM. In addition to these B-cell phenotyping antibodies, we dual-stained cells with the 444glyc-tet-AF647 and XBB- or BQ.1.1-tet-PE RBD-tetramers. We defined the IgD-IgM-CD19+ population as class-switched memory B cells and identified antigen-reactive B cells from this population using RBD-tetramer staining.

☒ Tick this box to confirm that a figure exemplifying the gating strategy is provided in the Supplementary Information.
